# Innate acting memory Th1 cells modulate heterologous diseases

**DOI:** 10.1073/pnas.2312837121

**Published:** 2024-06-05

**Authors:** Nikolas Rakebrandt, Nima Yassini, Anna Kolz, Michelle Schorer, Katharina Lambert, Eva Goljat, Anna Estrada Brull, Celine Rauld, Zsolt Balazs, Michael Krauthammer, José M. Carballido, Anneli Peters, Nicole Joller

**Affiliations:** ^a^Institute of Experimental Immunology, University of Zurich, 8057 Zurich, Switzerland; ^b^Department of Quantitative Biomedicine, University of Zurich, 8057 Zurich, Switzerland; ^c^Institute of Clinical Neuroimmunology, University Hospital, Ludwig-Maximilians-Universität München, 82152 Planegg, Germany; ^d^Novartis Biomedical Research, 4002 Basel, Switzerland; ^e^Biomedical Center, Faculty of Medicine, Ludwig-Maximilians-Universität München, 82152 Planegg, Germany

**Keywords:** adaptive immunity, T helper cells, infection, innate, autoimmunity

## Abstract

Through immune memory, infections have a lasting effect on the host. While memory cells enable accelerated and enhanced responses upon rechallenge with the same pathogen, their impact on susceptibility to unrelated diseases is unclear. We identify a subset of memory T helper 1 (Th1) cells termed innate acting memory T (T_IA_) cells that originate from a viral infection and produce IFN-γ with innate kinetics upon heterologous challenge in vivo. Activation of memory T_IA_ cells is induced in response to IL-12 in combination with IL-18 or IL-33 but is TCR independent. Rapid IFN-γ production by memory T_IA_ cells is protective in subsequent heterologous challenge with the bacterial pathogen *Legionella pneumophila*. In contrast, antigen-independent reactivation of CD4^+^ memory T_IA_ cells accelerates disease onset in an autoimmune model of multiple sclerosis. Our findings demonstrate that memory Th1 cells can acquire additional TCR-independent functionality to mount rapid, innate-like responses that modulate susceptibility to heterologous challenges.

## Significance

Through immune memory, infections leave lasting imprints on the host immune system. This is protective upon reinfection with the same pathogen, but whether and how this influences responses to unrelated challenges is mostly unclear. The present study identifies a subset of memory T helper 1 (Th1) cells arising after viral infection that can influence subsequent immune responses irrespective of their specificity. These innate acting memory Th1 cells are marked by preferential recruitment to sites of inflammation and rapid cytokine production upon challenge. As a consequence, they broadly modulate disease susceptibility and have a protective effect in subsequent infections while accelerating autoimmunity.

Disease susceptibility can be highly variable between individuals as highlighted by the broad range of disease courses seen, e.g., in the recent COVID-19 pandemic (1–3). Genetic predisposition influences the propensity of the immune system to respond to challenges as well as the magnitude of that response. In addition, environmental factors as well as interactions with pathogens and the microbiome contribute to the variability observed in disease susceptibility (4–9). Pathogen exposure triggers a transient effector response but also establishes a persisting pool of memory cells, that play an essential role in mediating long-term protection against secondary infections with the same pathogen (10). However, their impact on heterologous challenges is less clear. Although cross-reactive memory cells can alter the disease course in some settings (11, 12), their impact is restricted to very few specific combinations. Besides TCR-dependent activation, several in vitro studies have shown that memory T cells may also be activated in response to certain cytokine combinations through so-called bystander activation (13–16). Cytokine-mediated activation and IFN-γ production by CD8^+^ T cells are potently induced by IL-12 + IL-18 and to a lesser degree by IL-12 + TNFα (13, 17). Similarly, CD4^+^ T helper cells that display a memory phenotype can be activated in the absence of a TCR trigger in response to combinations of a STAT activator and an IL-1 family cytokine (16, 18–20).

Bystander activation of memory CD8^+^ T cells has been shown to influence disease severity in several disease settings including rheumatoid arthritis, hepatitis, and COVID-19 (21–23), but whether CD4^+^ T cells can play a similar role is unknown. Initial studies have started investigating whether in vitro generated CD4^+^ T cells or so-called memory phenotype CD4^+^CD44^+^ T cells present in naive mice may influence heterologous diseases (18, 24). However, whether and how classical memory CD4^+^ T helper cells established during prior infections influence the magnitude and more importantly the nature of subsequent immune responses to heterologous challenges in vivo is still unclear.

In this study, we investigated the response of virus-specific memory Th1 cells in heterologous challenges. To induce memory, we performed an acute infection with Lymphocytic Choriomeningitis virus (LCMV) and then rechallenged the mice with the unrelated bacterial pathogen *Legionella pneumophila* (Lpn) after the viral infection had been cleared. Even though the two pathogens do not harbor shared antigens, virus-specific memory CD4^+^ T cells mounted an early IFN-γ response upon bacterial challenge, which was sufficient to reduce the bacterial burden. The response of these innate acting memory T cells (T_IA_ cells) was TCR-independent and could be induced by cytokine stimulation alone. Furthermore, T_IA_ cells displayed a superior migratory capability that was essential for the protective effect observed in the bacterial challenge. In an autoimmune setting, the rapid, antigen-independent activation and enhanced migratory capacity of CD4^+^ T_IA_ cells enabled them to infiltrate the CNS and contribute to an earlier disease onset in a model of multiple sclerosis. Our findings thus uncovered a facet of memory CD4^+^ T cells in vivo, whereby they bear the potential to respond rapidly to heterologous challenges in a TCR-independent fashion that ultimately alters disease severity.

## Results

### Heterologous Protection from Infection.

**Fig. 1.**
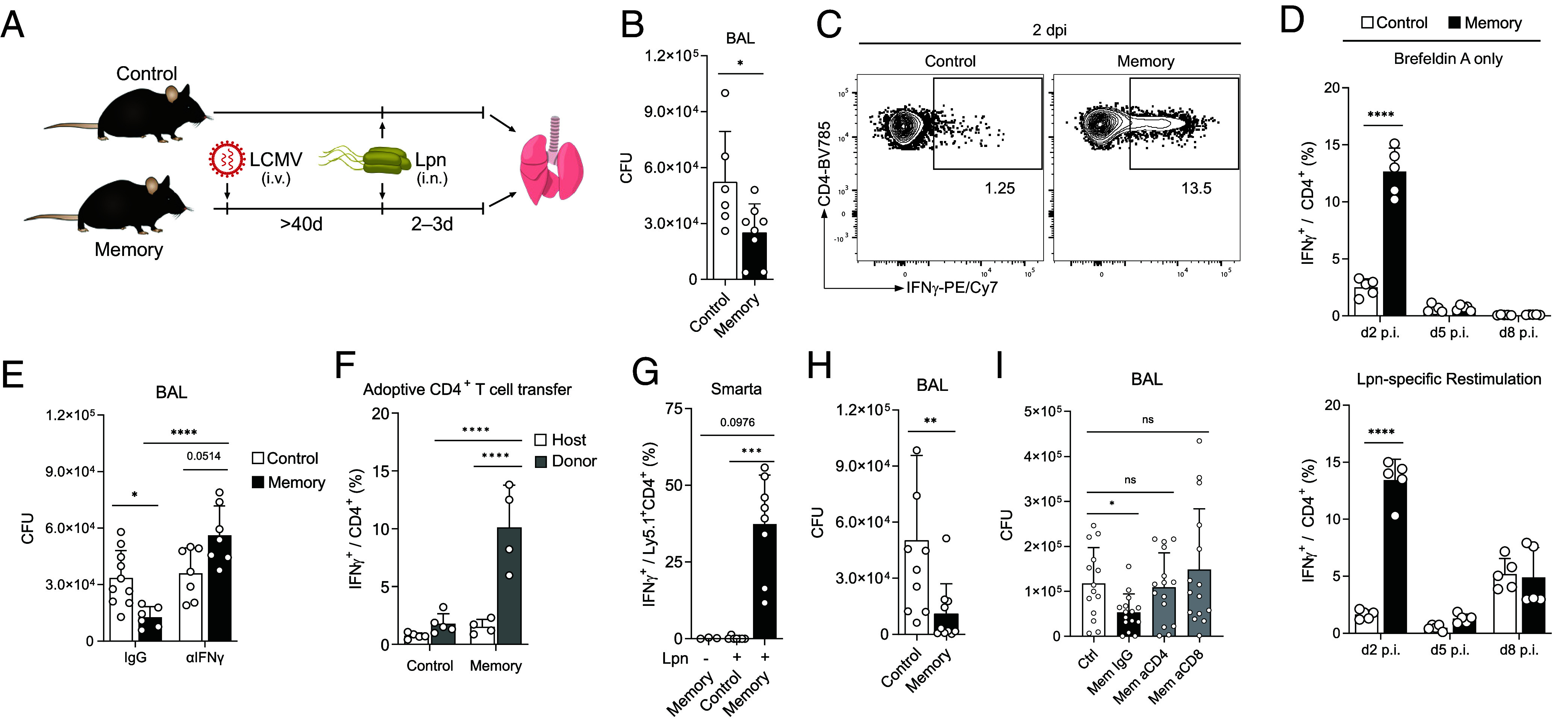
Early IFN-γ production in virus-experienced CD4^+^ T cells mediates protection against Lpn. Control and LCMV-experienced memory mice were challenged with Lpn. (*A*) Experimental layout of the heterologous infection model. (*B*) Bacterial titers from bronchoalveolar lavage (BAL) fluid 3 d post infection (dpi) (n = 6 to 8). (*C*) Representative FACS plot of the IFN-γ response of CD4^+^ T cells. (*D*) Time course of IFN-γ response with or without Lpn restimulation (n = 5). (*E*) Bacterial titers 3 dpi in mice treated intranasally on d0 and d1 with αIFN-γ neutralizing or IgG control antibody (n = 6 to 10). (*F*) 5 × 10^5^ CD4^+^ T cells isolated from the spleen were transferred i.v. 1 d before Lpn infection into congenic hosts and the IFN-γ response was measured 60 h post infection (n = 4 to 5). (*G*) Mice received 5 × 10^4^ SMARTA cells i.v. 1 d prior to LCMV infection (memory) or 10^6^ naive SMARTA cells 1 d before Lpn challenge (control). IFN-γ response was measured on day 2 post-Lpn challenge (n = 3). (*H*) Bacterial titers 3 dpi of *Rag2*^−/−^γ*c*^−/−^ mice that received 2 × 10^6^ naive or memory CD4^+^ T cells 2 d prior to Lpn infection (n = 9 to 10). (*I*) LCMV memory mice were treated with αCD4 or αCD8 depleting antibodies (or IgG isotype control) 5 and 3 d prior to Lpn infection. Bacterial titers were determined on day 3 post Lpn challenge (n = 14 to 15). Mean ± SD, Mann–Whitney *U* test (*B* and *H*), two-way ANOVA (*Šídák*; *D*–*F*), Kruskal–Wallis test (*G*), or Brown–Forsythe and Welch ANOVA (*I*).

To address whether and how memory T cells influence the outcome of unrelated challenges, we established a heterologous infection model using two antigenically distinct pathogens. We observed that prior LCMV infection conferred partial protection from a later bacterial challenge with Lpn as indicated by reduced bacterial titers 3 d post challenge when compared to a control group (Fig. 1 *A* and *B*). Using high dimensional CyTOF analysis to compare cell composition and function in the lungs of memory and control mice, we observed an increase in the proportion of neutrophils, as well as T and B cells in memory mice and a shift in IFN-γ production from NK toward T cells (*SI Appendix*, Fig. S1*A*). Interestingly, a prominent IFN-γ response was evident in the absence of antigen-specific restimulation in both CD4^+^ and CD8^+^ T cells from memory but not control mice, which was confirmed by classical flow cytometry (Fig. 1 *C* and *D* and *SI Appendix*, Fig. S1 *A–D*). These results are in line with previous studies reporting early bystander activation of CD8^+^ T cells in a number of settings (13, 22, 23). In addition, we also detected robust IFN-γ production in CD4^+^ T cells upon heterologous challenge in vivo, where previous studies were limited to in vitro settings without prior infection (24, 25).

Analysis of the T cell response over time revealed that like CD8^+^ T cells, CD4^+^ T cells from memory mice readily produced IFN-γ as early as 2 d post infection, which declined again thereafter (Fig. 1*D* and *SI Appendix*, Fig. S1 *C* and *D*). Importantly, this early IFN-γ production by CD4^+^ T cells was observed in the absence of pathogen-specific restimulation with Lpn and did not require any T cell–stimulating agents such as PMA/ionomycin or αCD3/αCD28 treatment (Fig. 1*D*). Additionally, this innate-like response was distinct from the antigen-specific T cell response toward Lpn detected on day 8, which was comparable between control and memory mice (Fig. 1*D* and *SI Appendix*, Fig. S1*D*). Furthermore, antigen-independent cytokine production was restricted to IFN-γ as neither TNF-α nor IL-17 could be detected at these early timepoints or in the absence of antigen-specific stimulation (*SI Appendix*, Fig. S1 *C* and *D*). Importantly, these CD4^+^IFN-γ^+^ T cells did not express markers for innate T cells such as NKT cells, γδ T cells, or MAIT cells (*SI Appendix*, Fig. S1*E*). This suggested that the early IFN-γ peak was dependent on memory Th cells generated during the earlier virus infection, while the second wave of IFN-γ was likely elicited by de novo priming upon Lpn infection. To confirm that IFN-γ is protective in this disease setting as previously reported (26), we blocked IFN-γ upon Lpn challenge and found that this abolished the heterologous protection (Fig. 1*E*), supporting the notion that the early antigen-independent IFN-γ secretion from memory T cells mediates the protective effect observed during the heterologous challenge.

To further investigate the mechanism of antigen-independent reactivation of CD4^+^ T cells, we first confirmed that the ability to rapidly produce IFN-γ upon heterologous challenge was not pathogen-specific and limited to LCMV-specific memory CD4^+^ T cells, as it also occurred after an initial vaccinia virus infection (*SI Appendix*, Fig. S1*F*). Rapid IFN-γ production upon heterologous challenge thus represents a common feature of memory CD4^+^ T cells. To determine whether this altered IFN-γ response was the result of a virus-experienced environment enabling the rapid cytokine production or of T cell–intrinsic features, we transferred memory (or control) CD4^+^ T cells into nave hosts before Lpn challenge and analyzed the innate-like response. This adoptive transfer revealed that the ability for early IFN-γ production was T cell–intrinsic as transferred memory but not control or endogenous CD4^+^ T cells secreted IFN-γ upon Lpn challenge (Fig. 1*F* and *SI Appendix*, Fig. S1*G*). Furthermore, transfer of Smarta CD4^+^ T cells, which carry a TCR specific for the LCMV gp61 peptide and do not react to Lpn antigens (*SI Appendix*, Fig. S1*H*), confirmed that IFN-γ production was not a consequence of cross-reactivity, since memory but not control Smarta T cells produced high amounts of IFN-γ 2 d after Lpn challenge and returned to baseline by day 5 (Fig. 1*G* and *SI Appendix*, Fig. S1*I*). Importantly, although memory CD8^+^ T cells also rapidly produced IFN-γ upon heterologous challenge (*SI Appendix*, Fig. S1*C*), transfer of CD4^+^ T cells from control vs. memory mice into *Rag2*^−/−^g*c*^−/−^ mice lacking T, B, and NK cells was sufficient to replicate the heterologous protection observed (Fig. 1*H*).

Finally, to determine the relative contribution of CD4^+^ and CD8^+^ T cells during the heterologous challenge, we depleted CD4^+^ or CD8^+^ T cells before Lpn challenge. While the number of IFN-γ^+^ CD8^+^ T cells did not change upon CD4^+^ T cell depletion, IFN-γ^+^ CD4^+^ T cell numbers were significantly increased in CD8^+^ T cell–depleted mice (*SI Appendix*, Fig. S1 *J*–*L*), suggesting that the IFN-γ response from the CD4^+^ T cell compartment does not exert its full potency in immunocompetent mice. Most importantly, both CD4^+^ and CD8^+^ T cell depletion abolished the heterologous protection and bacterial titers returned to those observed in control mice (Fig. 1*I*). Thus, both, CD4^+^ and CD8^+^ T cells play a nonredundant role in the heterologous protection observed and CD4^+^ memory T cells are not only capable but sufficient for conferring heterologous protection upon Lpn challenge. Viral infections therefore induce a CD4^+^ memory T cell population that can mount a rapid antigen-independent IFN-γ response. These innate acting memory CD4^+^ T cells (T_IA_ cells) can mediate heterologous protection by antigen-independent, early IFN-γ production when faced with an unrelated pathogenic challenge.

### Innate Acting Memory CD4^+^ T Cells.

**Fig. 2.**
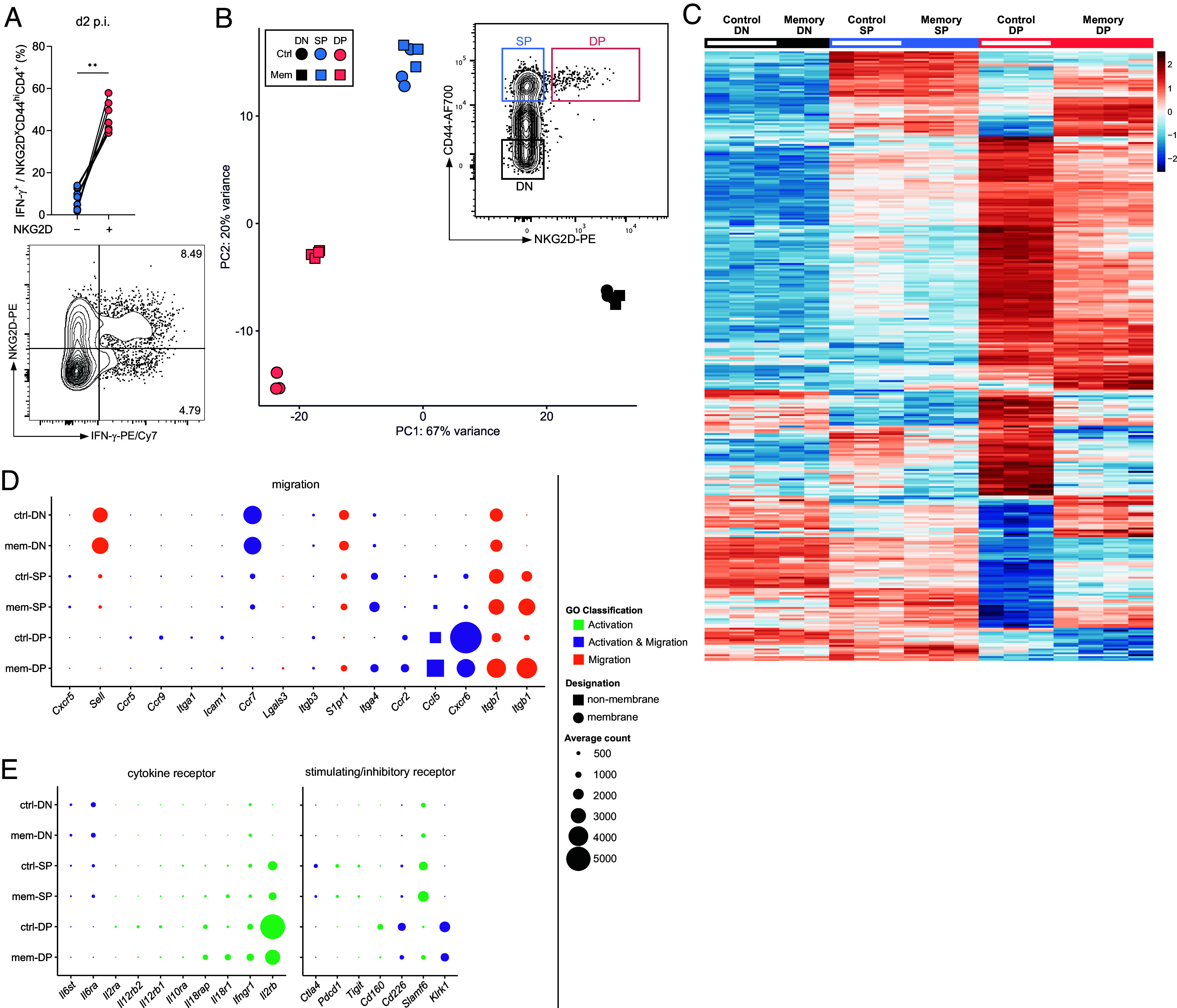
CD4^+^ T_IA_ cells have a distinct transcriptional profile. (*A*) IFN-g response in NKG2D^+^ and NKG2D^+^ CD44^+^CD4^+^ T cells (*Top*) and representative FACS plot gated on CD44^+^CD4^+^ T cells (*Bottom*) obtained from lungs of LCMV memory mice challenged with *Lpn* (2 dpi; n = 9, Wilcoxon matched-pairs signed-rank test). (*B*–*E*) RNA-seq analysis of CD4^+^ T cells isolated from spleens of control (Ctrl) and LCMV memory (Mem) mice. (*B*) Sorting strategy and principal component analysis (PCA) of the resulting populations. (*C*) Heatmap of genes in CD44^−^NKG2D^−^ (DN), CD44^hi^NKG2D^−^ (SP), and CD44^hi^NKG2D^+^ (DP) CD4^+^ T cells from LCMV memory and control mice (averaged normalized count from any population ≥10). (*D* and *E*) Differentially expressed genes (DEGs) relating to T cell migration (*D*) or activation (*E*) are highlighted. Balloon plots depicting average counts of the indicated genes for each cell population. DEGs are color-coded according to their GO classification. A curated list of migration- or cytokine receptor- and stimulatory/inhibitory receptor-related genes is shown.

To better characterize these CD4^+^ T_IA_ cells, we revisited our initial CyTOF analysis to look for markers that may distinguish CD4^+^ T_IA_ cells producing IFN-γ upon heterologous challenge from other memory CD4^+^ T cells. Such distinction would enable us to identify CD4^+^ T_IA_ cells at steady state even before they start secreting IFN-γ. Indeed, compared to IFN-γ^−^ CD4^+^ T cells, CD4^+^ T_IA_ cells showed higher expression of the germline-encoded receptor NKG2D (*SI Appendix*, Fig. S2*A*). NKG2D expression on CD4^+^ T cells has been associated with autoimmune disorders in mice and humans (22, 27, 28) and has been linked to bystander activation on CD8^+^ T cells (29). Classical flow cytometry confirmed that the NKG2D^+^CD4^+^ T cell fraction was highly enriched for IFN-γ^+^ cells and we found the CD44^+^NKG2D^+^CD4^+^ T cell fraction to be strongly expanded in memory mice (Fig. 2*A* and *SI Appendix*, Fig. S2 *B* and *C*), indicating that NKG2D can be used as a marker for CD4^+^ T_IA_ cells. We next compared the numbers of NKG2D^+^CD4^+^ T cells before and after Lpn challenge to determine whether CD4^+^ T_IA_ cells are already present in the lung before challenge or actively recruited to the site of infection. Memory, but not control mice showed a marked increase in NKG2D^+^CD4^+^ T cells in the lung accompanied by a decline in the spleen upon heterologous Lpn challenge (*SI Appendix*, Fig. S2 *D* and *E*), suggesting that CD4 T_IA_ cells are recruited from the spleen to the lung upon challenge. Indeed, blockade of T cell migration from secondary lymphoid organs using Fingolimod (FTY720) inhibited the increase in NKG2D^+^CD4^+^ T cell numbers in the lung and significantly reduced the number of IFN-γ^+^ CD4^+^ T cells upon Lpn challenge (*SI Appendix*, Fig. S2 *F* and *G*). The spleen thus appears to represent a reservoir for NKG2D^+^CD4^+^ T cells, which are recruited to peripheral sites upon heterologous challenge to produce IFN-γ.

Interestingly, control mice showed comparable numbers of splenic NKG2D^+^CD4^+^ T cells before Lpn infection but these were not recruited to the lung and could not mediate heterologous protection upon challenge (*SI Appendix*, Fig. S2 *D* and *E*). To dissect how CD4^+^ T_IA_ cells are able to respond to heterologous challenges at distant sites, we performed transcriptional profiling of splenic NKG2D^+^CD4^+^CD44^+^ T cells from memory and control mice and compared them to NKG2D^−^CD4^+^CD44^+^ memory as well as CD4^+^CD44^−^ naive T cells. NKG2D^+^CD4^+^CD44^+^ memory T cells from LCMV memory and control animals indeed formed distinct clusters, while CD4^+^CD44^−^ naive and NKG2D^−^CD4^+^CD44^+^ memory cells from the two animal groups were transcriptionally very similar (Fig. 2 *B* and *C* and *SI Appendix*, Fig. S3 *A* and *B*).

In line with a specific recruitment of memory CD4^+^ T_IA_ cells upon challenge, we observed differential expression of genes associated with cell migration when comparing NKG2D^+^CD4^+^CD44^+^ memory T cells from the two groups (e.g., *Itgb1*, *S1pr1*, *Itga4*) as well as genes up-regulated in NKG2D^+^CD4^+^CD44^+^ compared to NKG2D^−^CD4^+^CD44^+^ memory or CD4^+^CD44^−^ naive T cells (e.g., *Cxcr6*, *Ccr2*; Fig. 2*D*, and *SI Appendix*, Fig. S3 *B*–*D*). Furthermore, comparison between NKG2D^+^ and NKG2D^−^ CD4^+^CD44^+^ memory or CD4^+^CD44^−^ naive cells highlighted differential expression of a number of genes linked to T cell activation, including cytokine receptors (e.g., *Il2rb*, *Il18r1*) and stimulating/inhibitory receptors (e.g., *Cd226*, *Tigit*, *Pdcd1,* and *Klrk1* encoding for NKG2D; Fig. 2*E*, and *SI Appendix*, Fig. S3 *A*–*D*).

**Fig. 3.**
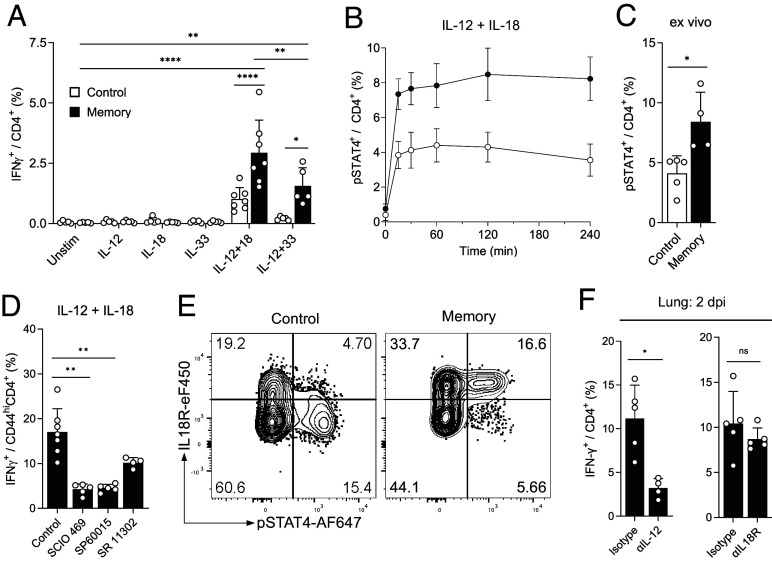
CD4^+^ T_IA_ cells can be activated through cytokines. Splenic (*A*, *B*, *D*, and *E*) or lung (*C* and *F*) CD4^+^ T cells from control and LCMV memory mice were analyzed by flow cytometry. (*A*) IFN-γ production after overnight stimulation with cytokines (n = 5 to 7). (*B* and *C*) pSTAT4 levels after in vitro stimulation (*B*, n = 4), and pSTAT4 levels ex vivo 2 dpi (*C*, n = 4 to 5). (*D*) Cytokine stimulated cells as in (*A*) treated with indicated inhibitors (n = 4 to 7). (*E*) Representative plots after 2 h stimulation with IL-12+IL-18, gated on CD44^+^CD4^+^ cells. (*F*) Ex vivo analysis after intranasal treatment with neutralizing αIL-12 or blocking αIL18R (or isotype control) (n = 4 to 5). Mean ± SD, ordinary two-way ANOVA (*Šídák*; *A*), Mann–Whitney *U* test (*C* and *F*), and Kruskal–Wallis test (Dunn; *D*).

Given that we found IFN-γ production by CD4^+^ T_IA_ cells to be TCR-independent (Fig. 1*G*), we first tested whether IFN-γ secretion in memory CD4^+^ T cells could be induced by cytokines alone and focused on cytokines for which we observed differential expression of the receptors as well as those induced upon Lpn infection (IL-12, IL-18). While no single cytokine was able to induce IFN-γ production from memory CD4^+^ T cells, combination of IL-12 + IL-18, which are both induced upon Lpn infection (30), and to a lesser degree IL-12 + IL-33, were able to stimulate IFN-γ secretion (Fig. 3*A* and *SI Appendix*, Fig. S4*A*). This is in line with a previous study reporting that IL-18 synergizes with IL-12 to produce IFN-γ in CD4^+^ T cells (16, 31). NKG2D^+^CD4^+^CD44^+^ T cells indeed showed higher expression of *Il12rb2* mRNA than NKG2D^−^CD4^+^CD44^+^ memory or CD4^+^CD44^−^ naive T cells (*SI Appendix*, Fig. S4*B*). Furthermore, CD4^+^ T cells from memory mice showed a higher responsiveness to IL-12 as indicated by higher STAT4 phosphorylation upon in vitro cytokine stimulation as well as upon Lpn infection in vivo (Fig. 3 *B* and *C*). Memory CD4^+^ T cells also expressed higher levels of IL-18R (but not the IL-33 receptor ST2 encoded by *Il1rl1*; *SI Appendix*, Fig. S4 *B* and *C*) and inhibition of the IL-18R signaling components p38 (SCIO469), JNK (SP60015), and AP-1 (SR 11302) reduced IFN-γ production by memory CD4^+^ T cells (Fig. 3*D* and *SI Appendix*, Fig. S4*D*). Finally, only CD4^+^ T cells from memory mice coexpressed the IL-18R on pSTAT4^+^ cells responding to IL-12 and were thus able to receive both signals necessary to induce IFN-γ production (Fig. 3*E*). In line with their ability to rapidly produce IFN-γ, CD4^+^ T_IA_ cells also express high levels of T-bet, both before and after challenge (*SI Appendix*, Fig. S4 *E* and *F*). Next, we addressed the importance of the identified cytokines for inducing IFN-γ production in CD4^+^ T_IA_ cells in vivo and focused on IL-12 and IL-18 as IL-33 could not be detected following Lpn infection (*SI Appendix*, Fig. S4*G*). Despite the ability of IL-18 to induce IFN-γ production in vitro, blockade of IL-18R in vivo only resulted in a slight reduction of IFN-γ that did not reach significance and the IL-18 signal may thus be compensated by other stimulatory factors in vivo (Fig. 3*F*). In contrast, IL-12 was essential for CD4^+^ T_IA_ cell activation in vivo as IL-12 blockade abolished the early IFN-γ response upon Lpn challenge (Fig. 3*F*). Cytokines alone are thus sufficient to activate CD4^+^ T_IA_ cells during a heterologous challenge in vivo and CD4^+^ T_IA_ cells require a combination of two cytokines for TCR-independent activation, whereby IL-12 is essential.

In line with the TCR-independent activation of CD4^+^ T_IA_ cells, 24 h stimulation with cytokines did not result in a strong upregulation of coinhibitory receptors, while stimulation of memory T cells through their TCR did induce such an upregulation (*SI Appendix*, Fig. S4*H*), as expected (32, 33). While in CD8^+^ T cells NKG2D can act as a costimulatory molecule and can also directly activate memory CD8^+^ T cells in a TCR-independent manner (34–36), NKG2D did not functionally contribute to the activation of memory CD4^+^ T_IA_ cells as stimulation with blocking or agonistic anti-NKG2D antibodies had no effect on the IFN-γ response (*SI Appendix*, Fig. S4*I*) and in vivo blockade with anti-NKG2D did not alter the heterologous response (*SI Appendix*, Fig. S4*J*). In line with these results, NKG2D expression is a poor predictor of the magnitude of the early, TCR-independent IFN-γ response (*SI Appendix*, Fig. S4*K*), despite the fact that NKG2D^+^CD4^+^CD44^+^ T cells were the most potent producers of IFN-γ upon cytokine stimulation (*SI Appendix*, Fig. S4*L*). Additionally, it is important to note that not all CD4^+^CD44^+^ memory T cells have this responsiveness to cytokine stimulation, as only a small fraction of memory CD4^+^ T cells were able to produce IFN-γ (*SI Appendix*, Fig. S4*L*). NKG2D thus acts as a marker rather than a functionally relevant receptor of CD4^+^ T_IA_ cells, which are activated in a TCR- and NKG2D-independent manner by cytokine alone whereby IL-12 is essential for their activation in vivo.

### Recruitment of CD4^+^ T_IA_ Cells.

**Fig. 4.**
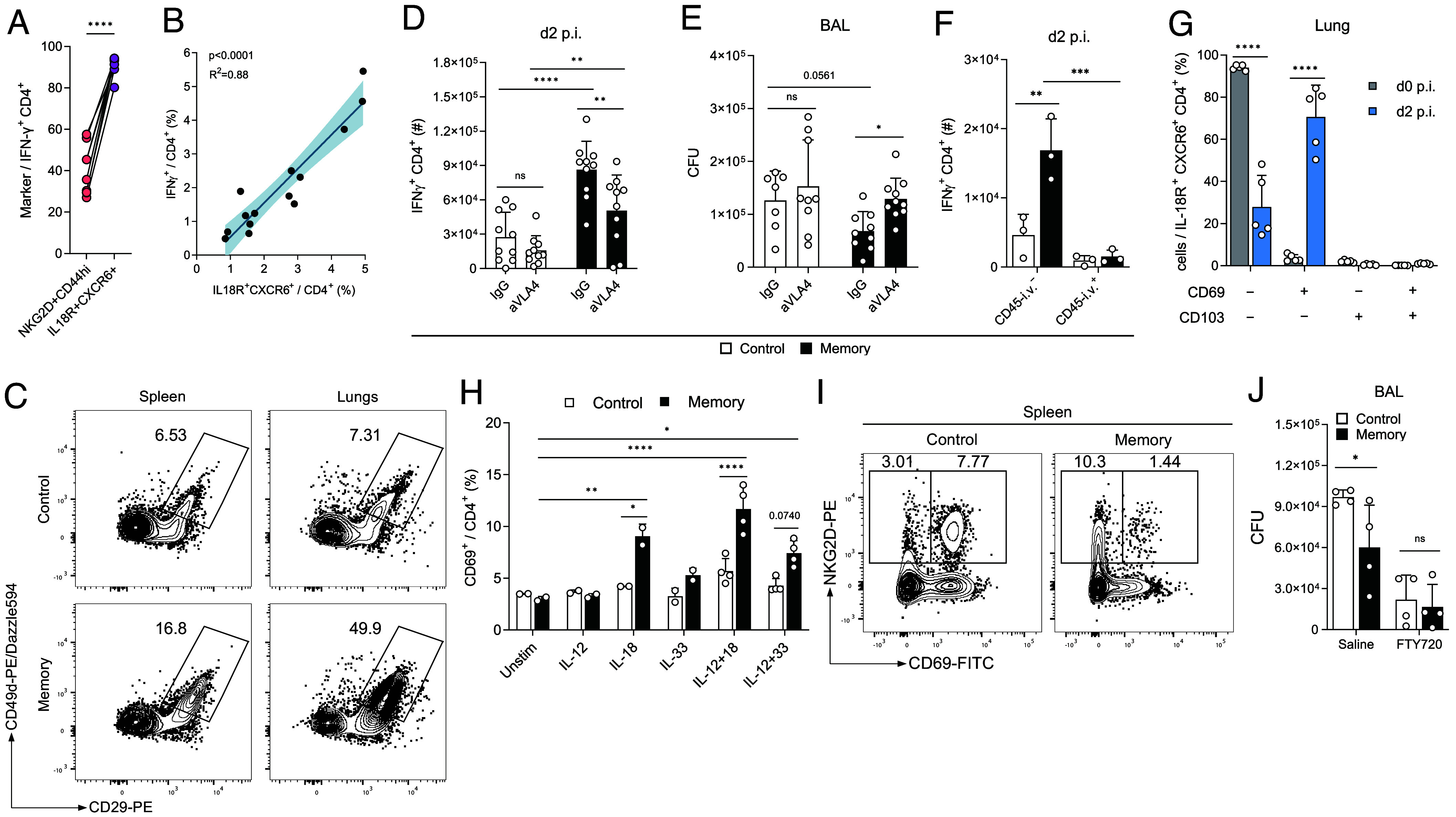
CD4^+^ T_IA_ cells have enhanced migratory capabilities. (*A*) Ex vivo expression of indicated markers in lung CD4^+^ T cells upon Lpn infection of LCMV memory mice (n = 10). (*B*) Linear regression of splenic control and memory CD4+ T cells stained ex vivo (*x* axis) and IFN-g production upon overnight IL-12+IL-18 stimulation (*y* axis). 95% CI is indicated (n = 14). (*C*) Representative FACS plots of integrin expression of Lpn challenged control vs. LCMV memory CD4^+^ T cells 2 dpi. (*D* and *E*) IFN-g response in lung CD4^+^ T cells (*D*) and Lpn titers (*E*) 2 dpi in mice treated with a-VLA4 blocking antibody or IgG control during Lpn challenge (n = 10). (*F*) Mice injected with aCD45 antibody i.v. prior to sacrifice (n = 3). (*G*) Expression of indicated markers among T_IA_ cells (n = 5). (*H*) CD69 expression in isolated splenic CD4^+^ T cells incubated with indicated cytokines overnight was determined by FACS (n = 2 to 4). (*I*) Representative FACS plot of splenic CD44^+^CD4^+^ T cells in control vs. LCMV memory mice pre-Lpn infection. (*J*) Lpn titers 3 dpi in mice treated with FTY720 or saline i.p. (n = 4). Mean ± SD, paired *t* test (*A*) and ordinary two-way ANOVA (*Šídák*; *D*–*H* and *J*).

To determine how the recruitment of CD4^+^ T_IA_ cells to the site of challenge is regulated, we further investigated molecules associated with T cell migration that were differentially expressed in NKG2D^+^CD4^+^CD44^+^ T cells from memory vs. control mice and in NKG2D^+^ vs. NKG2D^−^ CD4^+^CD44^+^ memory or CD4^+^CD44^−^ naive T cells (Fig. 2*D* and *SI Appendix*, Fig.s S3*D* and S5*A*). In line with the transcriptional data, we could observe a very high expression of the chemokine receptor CXCR6 on NKG2D^+^CD44^+^CD4^+^ T cells (*SI Appendix*, Fig. S5*A*) and IFN-γ^+^ CD4^+^ T_IA_ cells (*SI Appendix*, Fig. S5*B*). Indeed, CXCR6 together with IL-18R was an even better marker for IFN-γ expression than NKG2D (Fig. 4*A*), and additionally served as an excellent predictor of the magnitude of the IFN-γ response (Fig. 4*B*). Nevertheless, CXCR6 was highly expressed in CD4^+^ T_IA_ cells from memory mice but also in NKG2D^+^CD4^+^CD44^+^ T cells from control mice, which could be stimulated to produce IFN-γ in vitro, but were not recruited to the site of infection in vivo (*SI Appendix*, Figs. S2 *D* and *E* and S4*L*), hinting toward alternative recruitment mechanisms. Indeed, blocking of the CXCR6 ligand CXCL16 did not alter CD4^+^ T_IA_ cell recruitment or IFN-γ production. CXCR6 is thus not essential for migration of CD4^+^ T_IA_ cells to the site of infection (*SI Appendix*, Fig. S5*C*).

Besides CXCR6, CD4^+^ T_IA_ cells display high expression of the integrin VLA-4 (constituted of CD49d and CD29 encoded by *Itga4* and *Itgb1*; Figs. 2*D* and 4*C* and *SI Appendix*, Fig. S5 *A* and *D*), which plays an important role in lymphocyte homing and tissue entry (37). Indeed, blocking of VLA-4 resulted in reduced numbers of IFN-γ^+^ CD4^+^ T_IA_ cells and reverted bacterial titers to the level of control mice (Fig. 4 *D* and *E*), confirming the relevance of the VLA-4-dependent recruitment to the site of infection for T_IA_-mediated protection. To test whether CD4^+^ T_IA_ cells indeed preferentially enter the lung tissue upon Lpn challenge, we injected a fluorescently labeled anti-CD45 antibody intravenously shortly before sacrifice to distinguish cells in the vasculature and tissue of the lungs. Lpn-challenged memory mice indeed harbored more CD45-i.v. negative IFN-γ^+^ T cells that had entered the lung tissue than control mice (Fig. 4*F* and *SI Appendix*, Fig. S5*E*). The absence of CD45-i.v. labeling could be the consequence of tissue entry upon recruitment or indicate a previously established niche of tissue-resident memory CD4^+^ T cells in the lung. To investigate whether CD4^+^ T_IA_ cells exhibit tissue residency features, we analyzed their expression of the tissue residency markers CD69 and CD103 (38, 39). Analysis of CD69 and CD103 expression in the lung pre- and post-Lpn challenge revealed a lack of tissue residency marker expression among CD4^+^ T_IA_ cells prior to the Lpn infection (Fig. 4*G*). At day 2 post-Lpn infection, CD4^+^ T_IA_ cells were still negative for CD103, confirming that they do not represent a tissue-resident memory population. Nevertheless, the majority of CD4^+^ T_IA_ cells expressed CD69 after Lpn challenge. Upregulation of CD69 has been reported to occur upon tissue entry and it is speculated that local factors contribute to this induction (40). Because of the cytokine-mediated activation of CD4^+^ T_IA_ cells (Fig. 3 *A* and *F*), we wondered whether these cytokines could induce CD69 expression. Indeed, LCMV-experienced splenic CD4^+^ T cells, but not naive controls, showed an upregulation of CD69 upon overnight stimulation with IL-18 alone or in combination with IL-12 and to a lesser degree by stimulation with IL-12+IL-33 (Fig. 4*H*). These findings indicate that CD4^+^ T_IA_ cells can preferentially enter peripheral tissues where they then up-regulate CD69 and are retained upon cytokine activation.

Interestingly, both control and memory NKG2D^+^CD4^+^CD44^+^ T cells expressed elevated levels of S1PR1 (Fig. 2*D* and *SI Appendix*, Fig. S5*F*), a G-protein-coupled receptor required for lymphocyte egress from lymphoid organs (41). However, in contrast to NKG2D^+^CD4^+^CD44^+^ T cells from control mice, splenic CD4^+^ T_IA_ cells were negative for CD69 (Fig. 4*I*), which is known to promote T cell retention in the spleen and acts as a negative regulator of S1PR1 (42). The absence of CD69 together with a high expression of S1PR1 thus equips CD4^+^ T_IA_ cells with a superior ability to exit the spleen upon challenge (*SI Appendix*, Fig. S5*G*). Finally, complete blockade of T cell egress from secondary lymphoid organs using fingolimod abolished the early heterologous protection we observed (Fig. 4*J*), highlighting the importance of CD4^+^ T_IA_ cell migration for their protective function.

### CD4^+^ T_IA_ Cells Promote Autoimmunity.

**Fig. 5.**
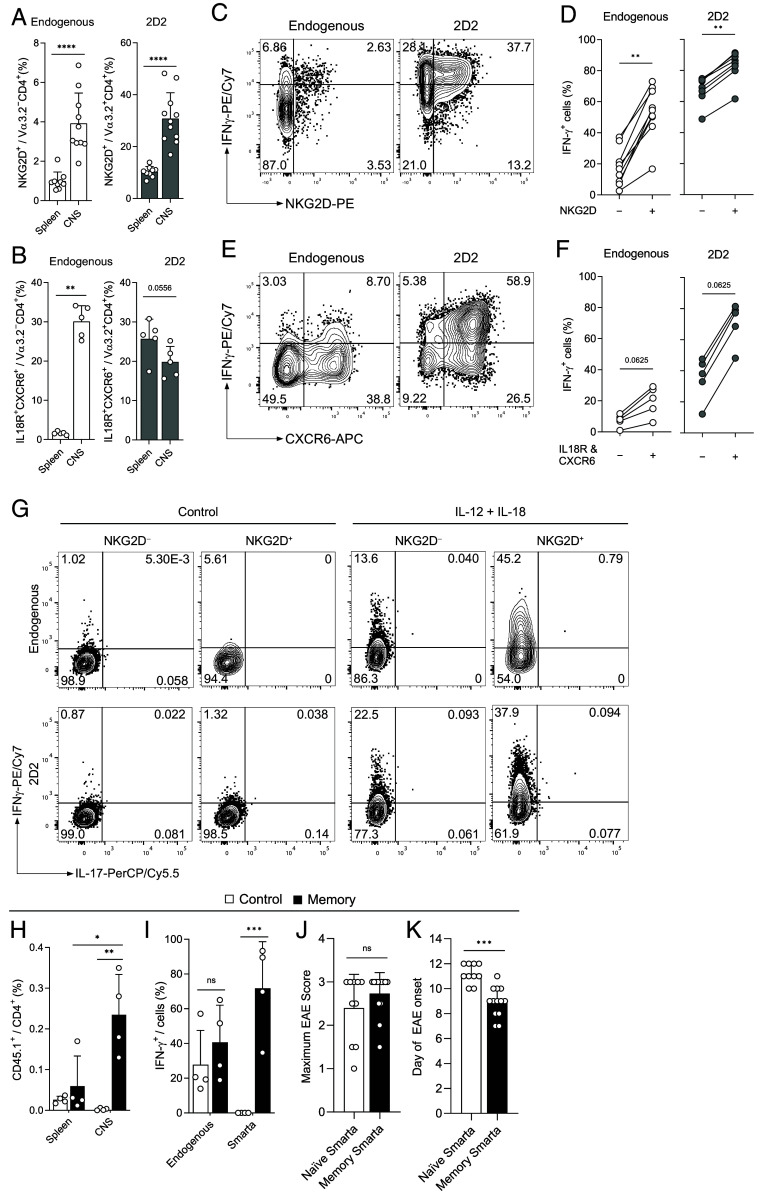
CD4^+^ T_IA_ cells promote CNS inflammation in EAE. For induction of EAE, Th1 polarized 2D2 cells were transferred into WT mice and identified by Vα3.2 expression (*A*–*F*). (*A* and *B*) Expression of NKG2D (*A*, n = 9 to 11) and IL-18R and CXCR6 (*B*, n = 5) in CD4^+^ T cells was determined by flow cytometry at the peak of EAE. (*C*–*F*) Representative plots (*C* and *E*) and summary graphs (*D*, n = 8 to 9, including samples from *F*; *F*, n = 5) of the IFN-γ response among NKG2D^−^ or NKG2D^+^ (*D*) and IL-18R^−^CXCR6^−^ or IL-18R^+^CXCR6^+^ (*E*) CD4^+^ T cells from the CNS at the peak of disease. (*G*) Cells isolated from the CNS were stimulated with IL-12+IL-18 or left untreated overnight and analyzed for IFN- production; plots are gated on CD4^+^ T cells. (*H* and *I*) MOG_35-55_/CFA-immunized C57Bl/6 mice received 2 × 10^6^ naive or memory Smarta CD4^+^ T cells (CD45.1^+^). Smarta frequency (*H*) and IFN-γ response in the CNS (*I*) (n = 4). (*J* and *K*) 5 × 10^5^ 2D2 CD4^+^ T cells and 5 × 10^5^ naive or memory Smarta cells were cotransferred into *Rag1*^−/−^ mice immunized with MOG_35–55_/CFA, monitored for disease and maximum disease score (*J*) and day of onset (*K*) were determined (n =10, pooled data from 2 out of 4 experiments). Mean ± SD, Mann–Whitney test (*A*, *B*, *J*, and *K*), Wilcoxon matched-pairs signed-rank test (*D* and *F*), and two-way ANOVA (*Šídák*; Hand *I*).

T cell migration to and cytokine production at tissue sites are not only essential in immunity to infections but also play an important role in autoimmune disorders. To determine whether CD4^+^ T_IA_ cells may contribute to the etiology of autoimmunity, we first assessed whether they are present in autoimmune settings. EAE is a well-established model for multiple sclerosis and can be induced by active immunization with CNS antigens or by adoptive transfer of activated T cells specific for CNS antigens such as 2D2 cells, which recognize a peptide derived from myelin (43, 44). Using the adoptive transfer EAE model, we found that CD4^+^ T_IA_ cell markers NKG2D^+^ or CXCR6^+^IL-18R^+^ were not only highly enriched on CNS antigen-specific 2D2 cells but also on nonspecific endogenous T cells, supporting the notion that CD4^+^ T_IA_ cells are also recruited to sites of autoimmune inflammation in an antigen-independent manner (Fig. 5 *A* and *B* and *SI Appendix*, Fig. S6 *A* and *B*). This is in line with reports that found increased frequencies of NKG2D^+^ CD4^+^ or CXCR6^+^ CD4^+^ T cells at the site of autoimmune inflammation in patients suffering from multiple sclerosis, rheumatoid arthritis, or systemic lupus erythematosus (22, 27, 45–48). Mirroring their effector function upon infectious challenge, NKG2D^+^ and CXCR6^+^IL-18R^+^ CD4^+^ T cells were higher producers of IFN-γ in active and passive models of EAE (Fig. 5 *C*−*F* and *SI Appendix*, Fig. S6 *C* and *D*). Importantly, CNS-infiltrating endogenous CD4^+^ T cells could be potently activated to produce high amounts of IFN-γ when stimulated with IL-12+IL-18 alone (Fig. 5*G*), confirming that true CD4^+^ T_IA_ cells capable of massive cytokine release in the absence of a TCR signal are indeed present at the site of autoimmune response.

Next, we tested our hypothesis that virus-experienced memory CD4^+^ T_IA_ cells are preferentially recruited to sites of autoimmune inflammation. To this end, we transferred naive or memory Smarta T cells, which do not recognize CNS antigens, into EAE recipient mice. In line with our hypothesis, we found that memory but not naive Smarta T cells infiltrated the CNS of EAE mice (Fig. 5*H*) and produced high amounts of IFN-γ, but not IL-17A, even in the absence of their cognate antigen (Fig. 5*I* and *SI Appendix*, Fig. S6*E*). Finally, while the cotransfer of LCMV-specific memory Smarta cells together with MOG-specific 2D2 cells into immunized *Rag1*^−/−^ mice did not impact disease severity when compared to naive Smarta cells (Fig. 5*J* and *SI Appendix*, Fig. S6 *F–H*), memory Smarta cells did accelerate EAE disease onset (Fig. 5*K*), confirming that CD4^+^ T_IA_ cells can contribute to the development of autoimmunity. Like in heterologous infectious challenges, CD4^+^ T_IA_ cells are thus preferentially recruited to the site of autoimmune inflammation, where they can be activated in a TCR-independent manner to produce IFN-γ and contribute to autoimmune disease.

## Discussion

The capability to generate memory cells upon pathogen encounter is one of the greatest advantages of the vertebrate immune system. Memory T cells mount an accelerated and augmented response upon reencounter of their cognate antigen resulting in enhanced pathogen control. Here, we show that memory CD4^+^ T cells generated in response to a viral infection are also capable of mounting an early IFN-γ response in unrelated heterologous challenges. This rapid and antigen-independent response exerted by CD4^+^ T_IA_ cells consequently modulates disease susceptibility in infectious and autoimmune settings.

Following heterologous bacterial challenge, CD4^+^ T_IA_ cells reduced the bacterial burden in a TCR-independent manner. Importantly, CD4^+^ T_IA_ cells alone were able and sufficient to confer this protection. CD4^+^ T_IA_ cells established following LCMV infection have a distinct transcriptional profile with up-regulated expression of cytokine and chemokine receptors such as IL-18R and CXCR6. Similar to memory CD8^+^ T cells (13), CD4^+^ T_IA_ cells can be stimulated in vitro to produce IFN-γ by the cytokine combination IL-12 + IL-18, both of which are present during Lpn infection. In vivo, IL-12 was essential for the CD4^+^ T_IA_ cell response. In contrast, we did not see a reduction of IFN-γ production by blocking signals through the IL-18R. This fits with our observation that IL-33 in combination with IL-12 was also able to stimulate IFN-γ secretion, although to a lesser degree. While IL-18 and IL-33 are both IL-1 family cytokines, IL-1β itself could not synergize with IL-12 to activate CD4^+^ T_IA_ cells. Whether this is due to the nature of the signal of each specific receptor or the ability of CD4^+^ T_IA_ cells to sense these cytokines remains to be determined. Furthermore, the possibility that the second signal could also be delivered through costimulatory molecules, such as, e.g., LPS (25), rather than from cytokines, has not been excluded. Thus, while IL-12 is essential for TCR-independent reactivation of CD4^+^ T_IA_ cells, a certain redundancy exists for the second signal necessary to evoke this heterologous response.

Interestingly, these cytokine combinations could also induce IFN-γ production in CD44^+^ T cells from control animals that to a certain degree resemble CD4^+^ T_IA_ cells and correspond to the previously described memory phenotype CD4^+^ T cells (18). However, despite their responsiveness to the cytokine stimulation, CD44^+^ T cells from control animals did not have the capacity to migrate to the site of heterologous challenge, which was crucial for the protective effect of CD4^+^ T_IA_ cells in memory mice. Although CXCR6 (together with IL-18R expression) serves as a good predictor of the IFN-γ response, it does not functionally contribute to CD4^+^ T_IA_ cell recruitment. This is in line with previous reports that found CXCR6 to be dispensable for CD4^+^ T cell migration to the site of inflammation (45, 49). Interestingly, a recent study revealed that the intestine forms a reservoir for Th17 cells from which pathogenic cells then disseminate via the spleen to sites of autoimmune inflammation (50). The reduction of CD4^+^ T_IA_ cell numbers in the spleen we observed following heterologous challenge suggests that the spleen may similarly serve as a kind of reservoir for Th1 CD4^+^ T_IA_ cells that can then be rapidly recruited to peripheral sites upon challenge. CD4^+^ T_IA_ cells are thus set apart from CD4^+^ memory T cells in control mice or previously described memory phenotype CD4^+^ T cells (18) by their ability to be recruited to the site of heterologous challenge where they then encounter a cytokine environment that allows for their TCR-independent activation and modulation of the immune response.

CXCR6^+^CD4^+^ T cells recently received much attention as prominent producers of cytokines and highly pathogenic effector cells during autoimmunity (45, 50, 51). While these studies focus on antigen-specific cells, we show here that CD4^+^ T_IA_ cells that are activated in a TCR-independent manner contribute to this pool of pathogenic cells and accelerate onset of autoimmune disorders, as we have seen for EAE. In line with our results, in vitro differentiated memory-like cells can contribute to EAE pathogenesis (24). Our study further expands this observation to classical memory Th1 cells as they are generated in the physiological setting of a viral infection. Viral infections have long been discussed as triggers for many autoimmune diseases and the rise in patients presenting with autoimmune symptoms following SARS-CoV-2 infections has strengthened the link between viruses and autoimmunity (52, 53). However, except for some rare instances (54), no causal link could be established between the virus and the autoimmune disease (55). Our study suggests that in addition to rare cases in which cross-reactive cells are established following viral infections (12), CD4^+^ T_IA_ cells generated as part of the virus-specific memory response are recruited to the site of autoimmune inflammation where they are activated to produce IFN-γ. Whether they have additional functional properties remains to be determined.

Collectively, our findings demonstrate that, upon challenge, memory CD4^+^ T cells are recruited to inflamed tissue sites and contribute to the local inflammatory response in an antigen-independent manner, thus favoring pathogen control but also promoting the onset of autoimmune pathology. This early, innate-like and antigen-independent nature of the T_IA_ cell response outlined in our study has highlighted an additional functionality of T cells to exert effector functions beyond the classical antigen-driven, delayed adaptive immune response.

## Limitations of the Study

In this study, we used transfers of CD4^+^ T cells into naive recipient mice to determine their capability to contribute toward autoimmune disease in the absence of confounding responses from endogenous cells. While these settings allow for studying the effects of isolated transferred adaptive immune cells and their potential in altering immune responses, they do not allow for a simultaneous assessment of the degree to which T_IA_ cells contribute to disease modulation under physiological conditions.

Furthermore, while our data revealed an essential role of IL-12 for T_IA_ cell activation, we observed some redundancy in the IL-1 family cytokine required, reflecting our finding that both IL-18 and IL-33 can serve as the second signal for their TCR-independent activation.

## Methods

### Mice.

C57BL/6 (B6) and Rag1-KO mice were purchased from Janvier Labs. Congenic Ly5.1 and Thy1.1, Smarta (56), 2D2 (44), and *Rag2*^−/−^γc^−/−^ (57) mice have been described previously. All animals were bred and housed in SPF and OHB facilities at LASC Zürich, Switzerland, or in the CAM in Munich, Germany. All experiments were performed in accordance with institutional policies and regulations of the relevant animal welfare acts and have been reviewed and approved by the Cantonal veterinary office or by the local animal ethics committee of the state of Bavaria (Regierung von Oberbayern) in accordance with European guidelines.

### Viruses, Bacteria, and Infections.

The LCMV WE strain was propagated on L929 cells and titrated on MC57G cells, and animals were infected i.v. with 200 FFU to induce an acute infection. Vaccinia virus was propagated on BSC40 cells, and mice were infected with 10^6^ PFU i.p. Then, 40 to 100 d after the primary infection, mice were intranasally infected with 3 × 10^6^ CFU *L. pneumophila* JR32 FlaA^−^ (Lpn) (58), grown on charcoal yeast agar plates. Animals were killed, perfused with PBS, and bronchoalveolar lavage (BAL), lung, and spleen were collected. For determination of bacterial titers, BAL and lungs from infected mice were collected, lungs were lysed using a Qiagen TissueLyser II, and samples were plated on charcoal yeast agar plates and grown for 3 d at 37 °C.

For the blockade of IL-18R or IL-12, Lpn was coadministered i.n. together with 20 µg anti-IL-18R antibody (clone 112624; R&D Systems) or 60 µg of anti-IL-12p40 antibody (clone C17.8; BioLegend). IFN-γ was neutralized by giving 100 µg anti-IFN-γ antibody (clone XMG1.2; BioLegend) i.n. on days 0 and 1 of the Lpn infection. To block the CXCR6 ligand of CXCL16, 100 µg of anti-CXCL16 antibody (clone 142417; R&D Systems) was injected i.v. FTY720 was injected daily at 1 mg/kg i.p until the mice were killed starting 1 d before Lpn infection. To deplete CD4^+^ or CD8^+^ T cells, mice were injected i.p. with 200 µg α-CD4 (GK1.5, BioLegend) or α-CD8 (YTS 169.4, BioXCell) antibody 5 d and 3 d prior to Lpn infection. For in vivo blockade of NKG2D, mice were injected with 200 µg α-NKG2D (CX5, BioXCell) or rat IgG1 i.p. on days −1, 0, and 1 of Lpn infection. For blockade of VLA-4, 100 µg α-VLA4 (PS/2, BioXCell) or Rat IgG2b were administered i.p. on days −1 and 0 of Lpn infection.

### Adoptive Cell Transfers.

For adoptive transfers and in vitro assays, CD4^+^ T cells were purified using MojoSort Mouse CD4 Nanobeads (BioLegend). For transfer into *Rag2*^−/−^g*c*^−/−^ mice, purified CD4^+^ T cells obtained from the spleen were additionally sorted for CD4 expression. To generate memory Ly5.1 Smarta cells, 10^4^ cells were adoptively transferred i.v. into B6 recipient mice 1 d prior to LCMV infection (200 FFU LCMV WE i.v.). Memory cells were obtained from spleens using the MojoSort Mouse CD45.1 selection kit (BioLegend) >40 d post infection.

To induce EAE by adoptive cell transfer, naive CD4^+^ T cells were isolated from the spleen and lymph nodes of 2D2 mice. To prepare a single-cell suspension, spleens and lymph nodes were mashed and passed through a 70 µm cell strainer. After erythrocyte lysis, naive CD4^+^ T cells were purified using the naive CD4^+^ T cell isolation kit (Miltenyi Biotec). Naive T cells were cultured at a concentration of 1.5 to 2 × 10^6^/mL in complete RPMI 1640 medium (supplemented with 10% heat-inactivated FBS, 1% penicillin–streptomycin, 10 mM HEPES, 2 mM L-glutamine, 1% nonessential amino acids, 1 mM sodium pyruvate, and 50 µM β-mercaptoethanol) in the presence of 7.5 to 10 × 10^6^/mL irradiated (35 Gy) splenocytes and 2.5 µg/mL soluble anti-CD3 antibody (clone 145-2C11, BioXCell). Th1 cells were generated by addition of IL-12 at a concentration of 10 ng/mL and anti-IL-4 antibody (clone 11B11, BioXCell) at a concentration of 10 µg/mL into the culture. For the generation of Th17 cells, naive T cells were cultured with IL-6 at a concentration of 30 ng/mL, TGF-ß at a concentration of 3 ng/mL, IL-1ß at a concentration of 20 ng/mL, and anti-IFN-γ (clone XMG1.2, BioXCell) and anti-IL-4 Ab (clone 11B11, BioXCell) at a concentration of 10 µg/mL. After 48 h, Th1 cells and Th17 cells were split with medium containing 10 ng/mL of IL-2 and medium containing 10 ng/mL of IL-23, respectively. All cytokines were purchased from BioLegend except IL-23 (Miltenyi Biotec). The different T cell subsets were analyzed for cytokine production after 4 d. After 5 to 8 d, cells were restimulated at a concentration of 2 × 10^6^/mL for 48 h in the presence of plate-bound anti-CD3 and anti-CD28 (clone PV-1; BioXCell) antibodies both at 2 µg/mL in fresh medium without any cytokines. A total of 2 to 4 × 10^6^ cytokine-producing cells were injected i.p. into B6 recipients.

For experiments using Smarta cells in the setting of EAE, either 2 × 10^6^ naive or memory Smarta cells were injected i.v. into C57BL/6 mice after MOG_35–55_/CFA immunization or 5 × 10^5^ naive or memory Smarta cells (together with 5 × 10^5^ 2D2 CD4^+^ T cells) were injected i.v. into RAG1 KO mice with MOG_35–55_/CFA immunization performed on the following day.

### Flow Cytometry.

FACS stainings were performed on single-cell suspensions from the spleen, lung, BAL, and CNS. Where indicated, mice received 2 µg anti-CD45.2 APC antibody (clone 104) in 200 µL PBS intravenously before they were killed. Three minutes following administration, they were killed by anesthesia (isoflurane) and cervical dislocation. The spleen samples were prepared by mechanical disruption in RPMI 1640 medium supplemented with 10% FCS, penicillin (100 IU/mL), and 1% L-glutamine. Lungs were enzymatically digested with collagenase D (Gibco) and DNase I (VWR) for 30 min, and immune cells were isolated using a 30% Percoll (GE Healthcare) gradient. Red blood cells were lysed with ACK buffer (155 mM NH_4_Cl, 10 mM KHCO_3_, and 0.1 mM Na_2_EDTA, pH: 7.4) for 3 min. For Lpn restimulation, cells were stimulated with Lpn-extract at 37 °C in 10% CO_2_ for 6 h before staining. When staining for intracellular cytokines, cells were incubated with Brefeldin A (BioLegend) for 4 h at 37 °C prior to staining. For surface stainings, antibodies were incubated for 20 to 30 min at RT in PBS. The Zombie NIR fixable dye (BioLegend) was used to exclude dead cells and debris. For intracellular cytokine staining, cells were permeabilized using the Cytofix/Cytoperm kit (BD Biosciences) for 5 to 8 min at RT, followed by antibody incubation for 20 to 30 min at RT. To stain for phosphorylated STAT4, cells were incubated for 12 min at 37 °C with PFA (4%) and upon washing fixed with 90% methanol for 30 min on ice. After fixation with methanol, cells were stained for 45 min at RT. For intranuclear staining, cells were fixed and permeabilized for 40 min at RT using Foxp3/Transcription Factor Staining Buffer Set (eBioscience), followed by antibody incubation for 20 to 30 min at RT.

For EAE experiments, cells were stimulated with PMA (50 ng/mL, Sigma-Aldrich) and ionomycin (500 ng/mL, Sigma-Aldrich) in the presence of monensin (0.7 µL/mL, GolgiStop; BD Biosciences) at 37 °C in 5% CO_2_ for 3.5 before staining. For surface stainings, antibodies were incubated for 20 to 30 min at 4 °C in PBS + 2% FBS. For intracellular cytokine staining, cells were fixed for 30 min at 4 °C with 0.4% paraformaldehyde (Merck KGaA) and permeabilized with PBS containing 2% FBS and 0.1% saponin (Sigma-Aldrich), followed by antibody incubation for 30 min at 4 °C. The Zombie UV fixable viability kit (BioLegend) was used to exclude dead cells and debris.

The following antibodies were used for flow cytometry: αCD4-BUV496 (RM4-5), αCD8a-BUV395 (53-6.7), αNK1.1-BUV615 (PK136), αPD-1-BUV737 (RMP1-30), αpSTAT4-AF647 (38/p-Stat4), and Streptavidin-BUV661 were purchased from BD. αCD45-eF450 (30-F11), αIL-17A-APC (eBio17B7), αIL-18Ra-PE, eFluor450 or PerCP/eFluor710 (P3TUNYA), streptavidin-APC, rat IgG1 κ-PE-Cy7 isotype control (eBRG1), rat IgG2α κ-PE isotype control (eBR2a) were all purchased from eBioscience. αCD11b-APC or AF700 (M1/70), αCD44-AF700 or PerCP (IM7), αCD4-BV605 or BV785 (RM4-5), αIFNγ-PE-Cy7 or PE/Dazzle594 (XMG1.2), αB220-PerCP-Cy5.5 (RA3-6B2), αVα3.2-FITC (RR3-16), αIL-10-PE (JES5-16E3), αCD19-PerCP-Cy5.5 (1D3), αCD45.1-FITC (A20), αCD45.2-PerCP-Cy5.5 (104), αCD29-PE (HMb1-1), αCD49d-PE/Dazzle594 (R1-2), αCD69 FITC (H1.2F3), biotin αCXCR6 (SA051D1), αNKG2D-PE (CX5), αNKG2D-PE/Cy7 (CX5), CCR2-APC (SA203G11), αIL-17A-PerCP-Cy5.5 or BV421 (TC11-18H10.1), αITGb7-FITC (FIB27), αT-bet-PE/Cy7 (4B10), αTCRβ-PE/Dazzle594 (H57-597), αTCRγδ-BB700 (GL3), αTigit-BV421 (1G9), αTNFa-PE/Cy7 (MP6-XT22), rat IgG2α κ-PerCP-Cy5.5 or APC isotype control (RTK2758), rat IgG2b κ-PE isotype control (RTK4530), rat IgG1 κ-PE isotype control (RTK2071) were all purchased from BioLegend. The MR1 tetramer technology was developed jointly by Dr. James McCluskey, Dr. Jamie Rossjohn, and Dr. David Fairlie, and the mouse MR1-5-OP-RU and control MR1-6-FP tetramer (both APC-labeled) were produced by the NIH Tetramer Core Facility as permitted to be distributed by the University of Melbourne.

FACSAria III was used for sorting of cells. Data were acquired on a BD LSR Fortessa, BD FACS Canto II, BD FACSverse, BD FACSymphony A5 analyzer (BD Bioscience), or Cytek Aurora and analyzed using FlowJo software (TreeStar).

### Cytokine Detection.

BAL fluid was collected with 1 mL PBS, and supernatants were stored at −80 °C. For detection of IL-12, IL-18, and IL-33 the mouse LEGENDplex kit (BioLegend) was used. The assay was performed following the manufacturer’s instructions. The bead-bound analytes were acquired on a Cytek Aurora following the manufacturer’s instructions and subsequently analyzed using LEGENDplex Data Analysis Software Suite (BioLegend).

### CyTOF.

Single-cell suspensions obtained from lung and spleens of control and LCMV-memory mice challenged with Lpn were incubated with Brefeldin A, labeled, and prepared for cytometry by time of flight (CyTOF) according to the manufacturer’s instructions and as previously described (59). In brief, all samples were stained with cisplatin (Fluidigm #20164; used to determine live cells), fixed with Fluidigm MaxPar® Fix I Buffer (Fluidigm #201067), and barcoded using the Fluidigm Cell-ID 20-Plex Pd Barcoding Kit (Fluidigm #201060). Subsequently, all barcoded lung and spleen samples were pooled into one lung and one spleen sample mix, respectively, and stained with the cocktail of monoisotope-labeled antibodies listed in *SI Appendix*, Table S1. Of note, the antibodies obtained from Fluidigm were purchased already labeled by the vendor, whereas the antibodies obtained from BioLegend were labeled in house using the specific Maxpar® antibody labeling kits from Fluidigm. Following antibody staining, the pools of lung and spleen samples were washed with MaxPar® Cell Staining Buffer (Fluidigm #201068), resuspended with Cell-ID™ Intercalator-Ir solution (Fluidigm #201192B; used to assess single-cell events) and left overnight at 4 °C. Next day, cells were washed again with MaxPar® Cell Staining Buffer, resuspended in MaxPar® water (Fluidigm #201069), pelleted and stored dry until acquisition. Immediately before data acquisition, the lung and spleen cell pellets were adjusted to 1 × 10^6^ cells/mL in MaxPar® water containing 10% EQ Four Element Calibration Beads (Fluidigm #201078; used to normalize data for signal variation occurring over acquisition time). Data acquisition was performed using a Fluidigm (Helios™) mass cytometer. Fcs data files were normalized with a software tool provided by Fluidigm and deconvoluted according to barcodes and analyzed in FlowJo.

### In Vitro Stimulation.

CD4^+^ T cells were isolated using the MojoSort Mouse CD4 T cell isolation kit (BioLegend). Upon isolation, cells were incubated with the indicated cytokines (10 to 100 ng/mL) for 12 to 16 h overnight. Inhibition of the MyD88-pathway was investigated by coincubating 1 µM SCIO 469 (TOCRIS), 5 µM SP600125 (Sigma), and 20 µM SR 11302 (TOCRIS) together with the IL-12 + IL-18 stimulation overnight. For in vitro antibody stimulation, flat bottom plates were coated with anti-CD3 (2 µg/mL, clone 145-2C11; BioXCell) and anti-CD28 (2 µg/mL, clone PV-1; BioXCell) or anti-NKG2D (10 µg/mL, clone CX5 or A10; BioLegend). Recombinant cytokines were purchased from BioLegend.

### RNA Sequencing.

Cells isolated from the spleen were sorted into 96-well plates (500 cells/well) by using a single-cell mask. For RNA isolation, the Smart-Seq2 protocol was applied as described in ref. 60. Briefly, Agencourt RNAClean XP paramagnetic beads (Beckman Coulter) were used in combination with a DynaMag-96 side skirted magnet (Thermo Fisher). cDNA was generated with the SuperScript II Reverse Transcriptase Kit (Thermo Fisher) and amplified with HiFi HotStart PCR Mix (KAPA Biosystems). For DNA clean-up, Agencourt AMPure XP beads were used (Beckman Coulter) as above. Nextera XT DNA sample preparation and index kits (Illumina) were used for preparation of libraries that were sequenced by the Functional Genomics Center Zurich (Zurich, Switzerland).

### Analysis of RNA Sequencing Data.

Raw sequencing files were aligned to the mouse genome (GRCm38) with HiSat2 (61) (version 2.2.1) following quality control with FastQC (https://www.bioinformatics.babraham.ac.uk/projects/fastqc/; version 0.11.9). Count tables were generated with featureCounts (62) (version 1.22.2) using the options ‘-t exon -g gene_id’ and the GTF file of the GRCm38 build (version 101) as reference. Data were analyzed using R (version 4.0.2). DESeq2 (63) (version 1.28.1) was used for normalization of counts and PCA. DEGs were defined by adjusted *P*-value < 0.05 and fold differences >2. Genes that had lower counts than 250 in all group averages were disregarded. Gene information together with gene ontology (GO) entries was obtained with Ensembl (version 101) using the biomaRt package (64, 65) (version 2.46.3). GO entries were used to group genes into the categories “activation” (GO entries: “activation” OR “immune response” OR “cytokine” OR “positive regulation of cell cycle”), “migration” (GO entries: “taxis” OR “migration” OR “chemokine” OR “cell adhesion”), “activation & migration” (activation AND migration), “transcription factor” (GO entry: “DNA-binding transcription factor activity”), and “regulator of transcription factor” (GO entry: “regulation of DNA-binding transcription factor activity”). Furthermore, genes were distinguished by their association with cell membrane/being at the cell surface (“integral component of membrane” OR “cell surface” OR “anchored component of plasma membrane”). To plot the results, the packages ggplot2 (66) (version 3.3.3), pheatmap (version 1.0.12), UpSetR (67) (version 1.4.0), and VennDiagaram (version 1.6.20) were used.

### Quantitative RT-PCR.

CD4^+^ T cells were isolated from the spleen using the MojoSort Mouse CD4 T cell isolation kit (BioLegend) and sorted according to their expression of CD44 and NKG2D. After the sort, cells were taken up in Buffer RLT (Qiagen) and stored at −20 °C. For RNA extraction, the RNeasy kit (Qiagen) was used by following the manufacturer’s instructions. Following the extraction, cDNA was created using the High-Capacity cDNA Reverse Transcription Kit (Applied Biosystems). For measurement of relative gene expression, real-time quantitative PCR (RT-qPCR) was performed using TaqMan Fast Advanced Master Mix (Applied Biosystems) and the following primers which were all purchased from Applied Biosystems: *Actb* (Mm00607939_s1), *Il12rb2* (Mm01183807_m1), *Il18r1* (Mm00515178_m1), *Il1rl1* (Mm00516117_m1), and *S1pr1* (Mm02619656_s1). All measurements were acquired on the Bio-Rad CFX384 Touch Real-Time PCR Detection System, and cycle threshold values were obtained through the CFX Maestro software (Bio-Rad).

### EAE Induction and Scoring.

B6 mice were immunized with 100 to 200 µg MOG_35–55_ (BioTrend) emulsified in CFA (Difco Laboratories) containing 5 mg/mL *Mycobacterium tuberculosis* (Difco Laboratories). Additionally, they received 150 ng PT (List laboratories) on days 0 and 2 after immunization.

To induce EAE in RAG1 KO mice, CD4^+^ T cells were isolated from the spleen and lymph nodes of 2D2 mice. To prepare a single-cell suspension, spleens and lymph nodes were mashed and passed through a 70 µm cell strainer. After erythrocyte lysis, CD4^+^ T were purified using magnetic beads coated with anti-CD4 antibody (clone L3T4) according to the manufacturer’s instructions (Miltenyi Biotec). A total of 5 × 10^5^ 2D2 CD4^+^ T cells were injected i.v. into RAG1 KO mice. On the next day, the animals were immunized with 30 µg MOG_35-55_ emulsified in CFA and received 150 ng PT on days 0 and 2 after immunization.

Animals were monitored daily for the development of classical and atypical signs of EAE according to the following criteria: 0, no disease; 1, decreased tail tone or mild balance defects; 2, hind limb weakness, partial paralysis, or severe balance defects that cause spontaneous falling over; 3, complete hind limb paralysis or very severe balance defects that prevent walking; 4, front and hind limb paralysis or inability to move body weight into a different position; and 5, moribund state.

### Isolation of Mononuclear Cells from the CNS.

Recipient mice were killed at the peak of disease and perfused through the left cardiac ventricle with PBS. The brain and spinal cord were cut into pieces and digested for 30 min at 37 °C with collagenase D (3.75 mg/mL; Roche) and DNase I (1 mg/mL; Sigma-Aldrich). To prepare a single-cell suspension, the tissues were mashed and passed through a 70 µm cell strainer. Mononuclear cells were isolated by a Percoll gradient (70%/37%) centrifugation (GE Healthcare).

## Statistics.

All statistical analyses, with the exception of RNAseq data, were performed using GraphPad Prism and were two sided. Outliers were identified for the following data using GraphPad Prism’s ROUT (2%) method: Fig. 1*C* (Control aIFNγ: 118800; Memory aIFNγ: 149760, 150240), Fig. 4 *A*, *Left* (Spleen: 4.47, 3.09; CNS: 15.80), Fig. 4 *A*, *Right* (Spleen: 24.30, 24.50), *SI Appendix*, Fig. S1*E* (Control: 5.25), Fig. 1*G* (Control: 16.7), *SI Appendix*, Fig. S2*C* (Memory d2: 351688), *SI Appendix*, Fig. S2*F* (Control FTY720: 242084.584), *SI Appendix*, Fig. S4*D* (IL-18 d0-Memory: 7.74; IL-18 d5-Memory: 59.67; IL-33 d2-Control: 63.54), *SI Appendix*, Fig. S4*F* (Memory: 77.1), *SI Appendix*, Fig. S4*J* (aNKG2D: 24.7), *SI Appendix*, Fig. S5*F* (Memory Spleen: 28.4), *SI Appendix*, Fig. S6 *B*, *Middle* (NKG2D^+^: 6.58 and subsequently its pair), and *SI Appendix*, Fig. S6*E* (Smarta Control: 62.5). Data with sample size <10 were analyzed using nonparametric tests. Data with sample size ≥10 were tested for normality with the Shapiro–Wilk test and Q–Q plot analysis. Statistical significance is defined as *P* < 0.05 and shown as *P < 0.01 as **P < 0.001 as ***P < 0.0001 as ****. *P*-values between 0.10 and 0.05 are indicated by the exact value.

## Supplementary Material

Appendix 01 (PDF)

## Data Availability

Source data for all figures and supplementary figures are provided with the paper. Sequencing has been deposited on the ArrayExpress database at EMBL-EBI (www.ebi.ac.uk/arrayexpress) and is available via accession number E-MTAB-11521 (68). The code used to analyze the RNA sequencing data can be found at https://github.com/nimayassini/Early_Responder_Memory_CD4_Tcell_2022 (69). Correspondence and requests for materials should be addressed to Nicole Joller (nicole.joller@uzh.ch).
