# Supplementary material for: Innate acting memory Th1 cells modulate heterologous diseases
Source: Proc Natl Acad Sci U S A. 2024 Jun 5;121(24):e2312837121. doi: 10.1073/pnas.2312837121 (PMC11181110; doi:10.1073/pnas.2312837121)
Supplement: Supplementary file 1 — Appendix 01 (PDF) [file pnas.2312837121.sapp.pdf]

PNAS

Supporting Information for
Innate acting memory Th1 cells modulate heterologous diseases

Nikolas Rakebrandt*, Nima Yassini*, Anna Kolz, Michelle Schorer, Katharina Lambert, Eva Goljat, Anna Estrada Brull, Celine Rauld, Zsolt Balazs, Michael Krauthammer, José M. Carballido, Anneli Peters, Nicole Joller

Nicole Joller
Email: nicole.joller@uzh.ch

This PDF file includes:

Figures S1 to S6
Table S1

Other supporting materials for this manuscript include the following:

Datasets S1 (E-MTAB-11521 on the ArrayExpress database at EMBL-EBI)

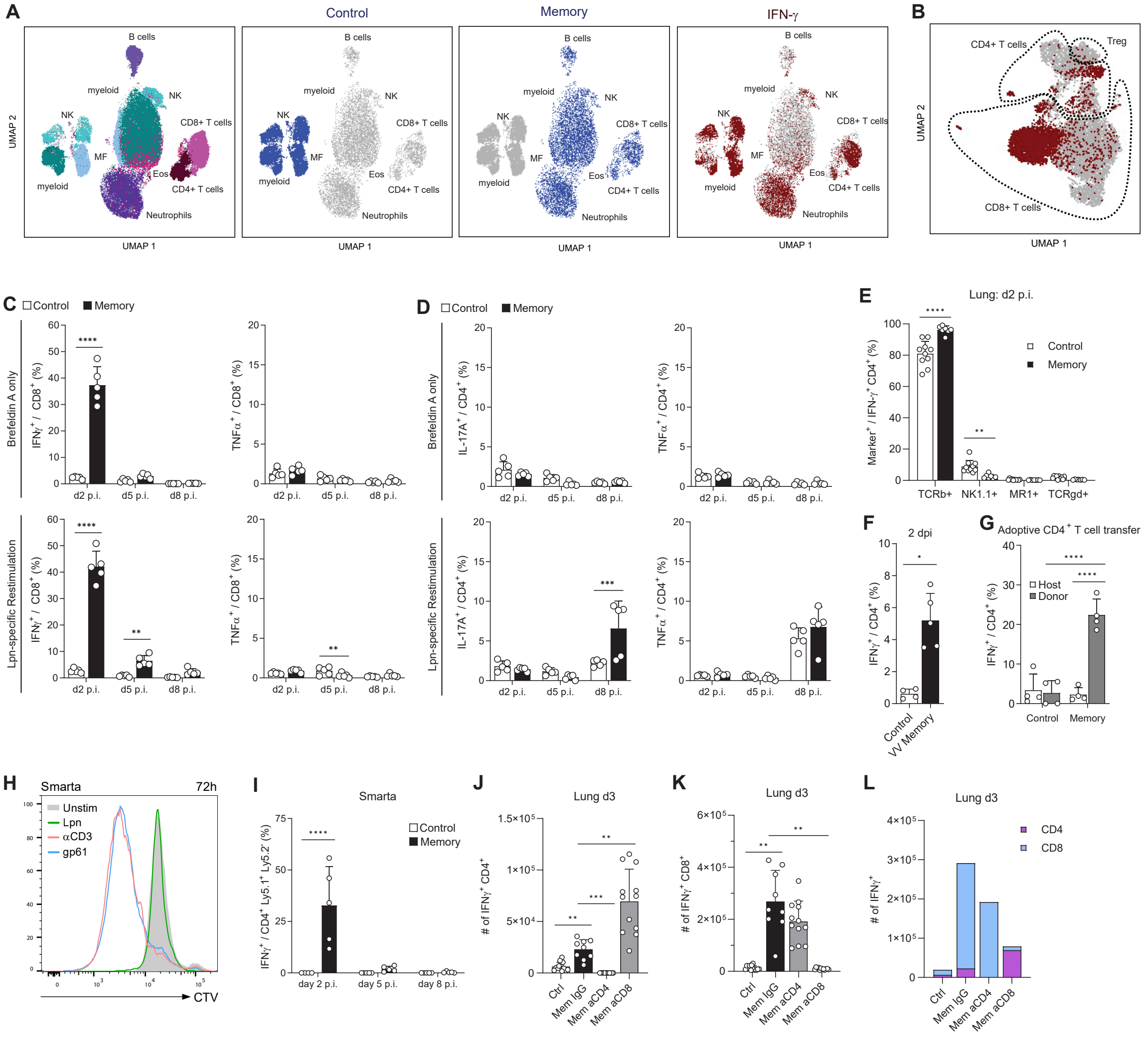

Fig. S1. Early antigen-independent response in virus-experienced T cells upon heterologous infection. Control and LCMV memory mice were challenged with Lpn (A-E, I-L). Cells from the lung were either treated with Brefeldin A only (A-G, H-K) or were restimulated with Lpn-extract followed by Brefeldin A incubation (C-D). A, UMAP of the combined dataset acquired by CyTOF (days 0 and 3; n = 3). Color code indicates manual annotation according to lineage marker expression profiles (left) or the indicated subset. B, same as (A), gated on T cells, IFN-γ^{+} cells are highlighted. C-D, Cytokine response from CD8^{+} (C) and CD4^{+} (D) T cells was measured by flow cytometry (n = 5). E, CD4^{+}IFN-γ^{+} cells were examined for their expression of indicated markers (n = 7-10). F, IFN-γ production of control or vaccinia virus (VV)-experienced CD4^{+} T cells on day 2 after Lpn challenge (n = 4-5). G, 5x10^{5} CD4^{+} T cells isolated from the lung were transferred i.v. into congenic hosts one day before Lpn infection and the IFN-γ response was measured 2 days post infection (n = 4-5). H, Splenocytes from naive Smarta mice were isolated, CellTrace Violet (CTV) labelled and stimulated as indicated for 3d. I, mice received 5x10^{4} Smarta cells i.v. one day prior to LCMV infection (memory) or 10^{6} naïve Smarta cells one day before Lpn challenge (control). IFN-γ response in SMARTA cells was determined on the indicated days (n = 5). J-L, Mice received αCD4 or αCD8 depletion or IgG control antibody 5 and 3 days prior to Lpn infection (n = 9–12). Number of IFN-γ^{+} CD4^{+} T cells (J, L) and IFN-γ^{+} CD8^{+} T cells (K, L) was determined on day 3. Mean ± s.d., Brown-Forsythe and Welch ANOVA (J, K), two-way ANOVA (Šídák; C, D, E, G, I) and Mann-Whitney U test (F).

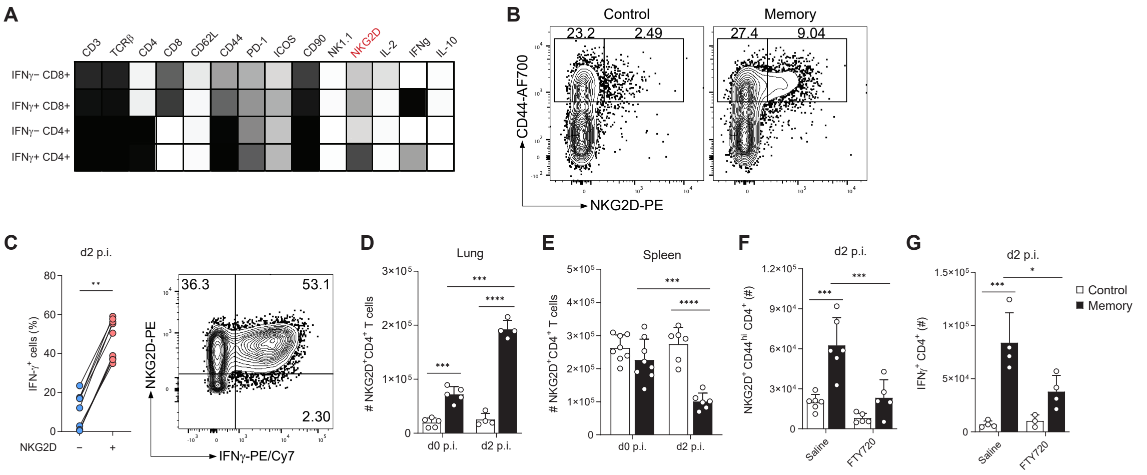

Fig. S2. Increased numbers of NKG2D$^+$ CD44^{hi} CD4$^+$ T cells in the lung are dependent on migration. A, cells from the lung of LCMV memory or control mice challenged with Lpn were treated with Brefeldin A and acquired by CyTOF. T cell populations were annotated according to lineage marker expression and a heat map showing relative expression of the indicated markers within the respective T cell populations is shown. B, representative FACS plots of CD4$^+$ T cells from the lung of LCMV memory or control mice 2d post Lpn infection. C, IFN-γ response in NKG2D$^-$ and NKG2D$^+$ CD44$^+$CD8$^+$ T cells (left) and representative FACS plot gated on CD44$^+$CD8$^+$ T cells (right) obtained from lungs of LCMV memory mice challenged with Lpn (2 dpi; n=9). D–E, NKG2D$^+$ CD44$^+$CD4$^+$ T cells in LCMV memory or control mice were analyzed before (d0) and 2d after Lpn challenge (n = 4–8). F–G, total numbers of the indicated cell populations from lungs of mice treated with FTY720 (20 mg i.p.) or PBS (n = 3–6). Mean ± s.d., statistical significance was tested by Wilcoxon matched-pairs signed rank (C), and two-way ANOVA (Šídák).

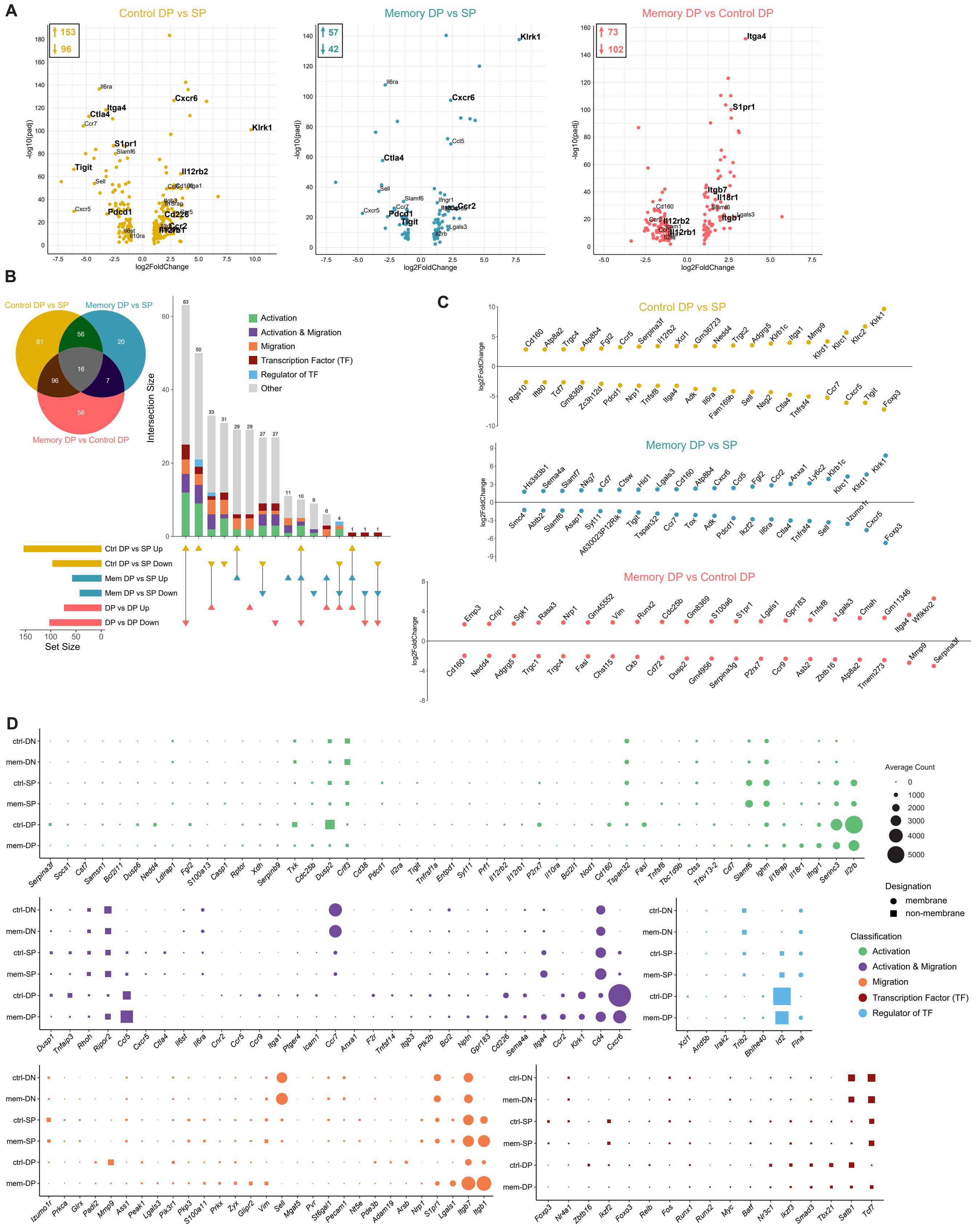

Fig. S3. Transcriptional analysis reveals distinct profiles in CD44^{hi}NKG2D^{+} CD4^{+} T cells. A, fold-change vs.

P value (volcano) plot of gene expression in the three indicated paired samples (DN: $CD44^{-}NKG2D^{-}$, SP: $CD44^{hi}NKG2D^{-}$, DP: $CD44^{hi}NKG2D^{+}$ $CD4^{+}$ T cells). B–D, differentially expressed genes (DEGs) from RNA-Seq data were obtained by combining 3 pairwise comparisons. Venn diagram shows the number and overlap of DEGs obtained from each comparison. B, UpSet plot depicts up- and down-regulated genes and the overlap among them. Color-coded vertical bars represent number of DEGs classified by their GO term. C, top 20 up- and down-regulated genes within each pairwise comparison. DEGs from each paired analysis was combined and subdivided according to their GO classification. D, balloon plot shows the average normalized counts of each cell population for the indicated genes.

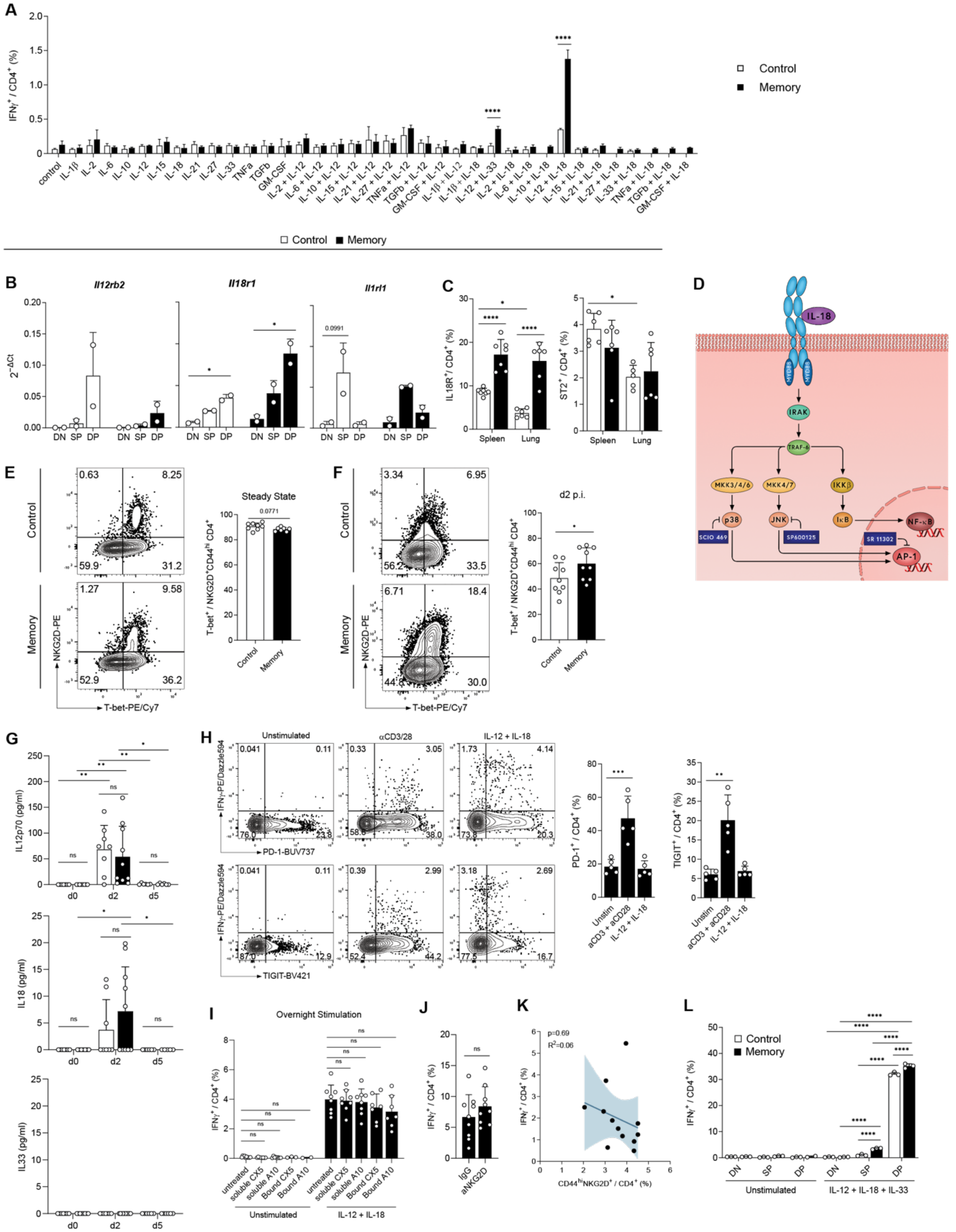

Fig. S4. TCR-independent activation of CD4+ T_{IA} cells through cytokine stimulation. A–B, CD4+ T cells isolated from spleen of LCMV memory or control mice were incubated with the indicated cytokines for 16h and analyzed by flow cytometry (A, n = 3–4). Cells were sorted according to the gating shown in Figure 2B and relative gene expression was determined by qRT-PCR (B, n = 2). C, *ex vivo* expression of the indicated markers in CD4+ cells from LCMV memory or control mice (n = 5–7). D, scheme depicting IL-18R signaling and intervention points of the

inhibitors used in Figure 3D. E–F, T-bet expression of T_{IA} cells in the spleen at steady state (E, n = 8) and in the lung 2d post Lpn challenge (F, n = 9). Representative FACS plots were gated on $CD4^+CD44^+$ cells. G, Bronchoalveolar Lavage (BAL) fluid was collected on the indicated days and IL-12, IL-18, and IL-33 concentrations were measured by CBA (n = 5–10). H–I, splenic $CD4^+$ T cells from LCMV memory mice were treated as indicated for 24h (H, n = 3) or overnight (I, n = 2–10; Clone CX5: antagonistic α-NKG2D, Clone A10: agonistic α-NKG2D; added in solution or plate bound as indicated) before IFN-γ production was determined by flow cytometry. J, d2 response of $CD4^+$ T_{IA} cells in the lung of LCMV memory mice treated with blocking α-NKG2D (CX5) antibody or IgG control on d−1, d0, and d1 of Lpn infection (n=9). K, linear regression of splenic control and memory $CD4^+$ T cells stained *ex vivo* (x-axis) and IFN-γ production upon overnight IL-12+IL-18 stimulation (y-axis). 95% confidence interval is indicated (n = 12). L, using the sorting strategy from Figure 2B, splenic $CD4^+$ T cells were treated overnight as indicated (n = 2–3). Mean ± s.d., two-way ANOVA (*Šídák*; A–D, I, L), Mann-Whitney U test (F, G, J) and Kruskal-Wallis test (H).

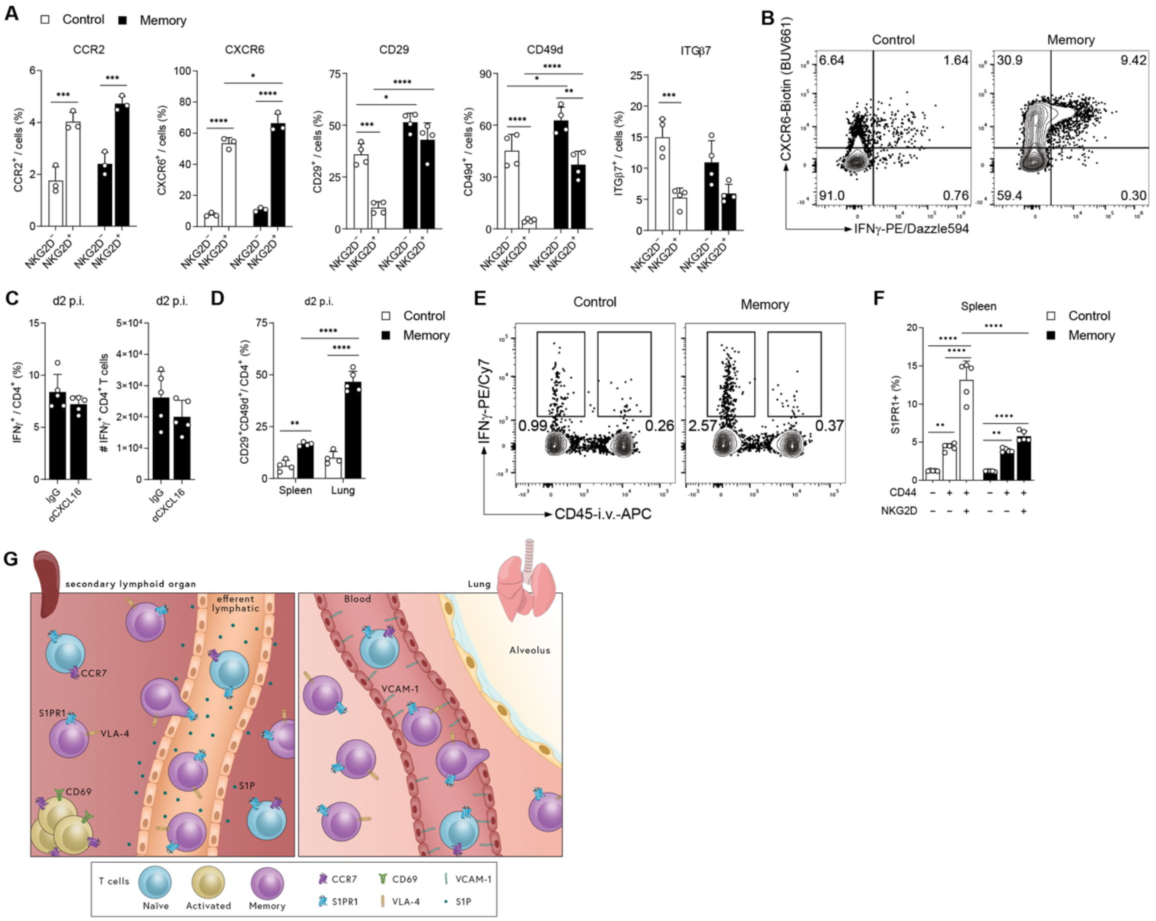

Fig. S5. Migration-associated markers expressed in CD4 T_{IA} cells. A, frequencies of the indicated chemokine receptors and integrins among NKG2D$^+$ or NKG2D$^-$ CD44^{hi}CD4$^+$ T cells from control or LCMV memory mice (n = 3–4). B, *ex vivo* FACS staining of CD4$^+$ T cells from the lung at d2 post Lpn infection. C, *ex vivo* analysis of lung CD4$^+$ T cells from LCMV memory mice d2 post Lpn infection after treatment with blocking αCXCL16 antibody or control IgG (n = 5). D, frequency of VLA-4$^+$ cells among CD4$^+$ T cells (n = 4–5). E, IFN-γ production in lung CD4$^+$ T cells was assessed 2d after Lpn challenge in mice injected with αCD45 Ab i.v. prior to sacrifice to stain leukocytes within the vasculature. F, S1PR1 frequency among indicated populations pre-Lpn challenge determined by flow cytometry (n=5). G, schematic representation of S1PR1-dependent lymphocyte egress and VLA-4 mediated extravasation. Mean ± s.d., two-way ANOVA (Šídák; A, E, G) and Mann-Whitney U test (C).

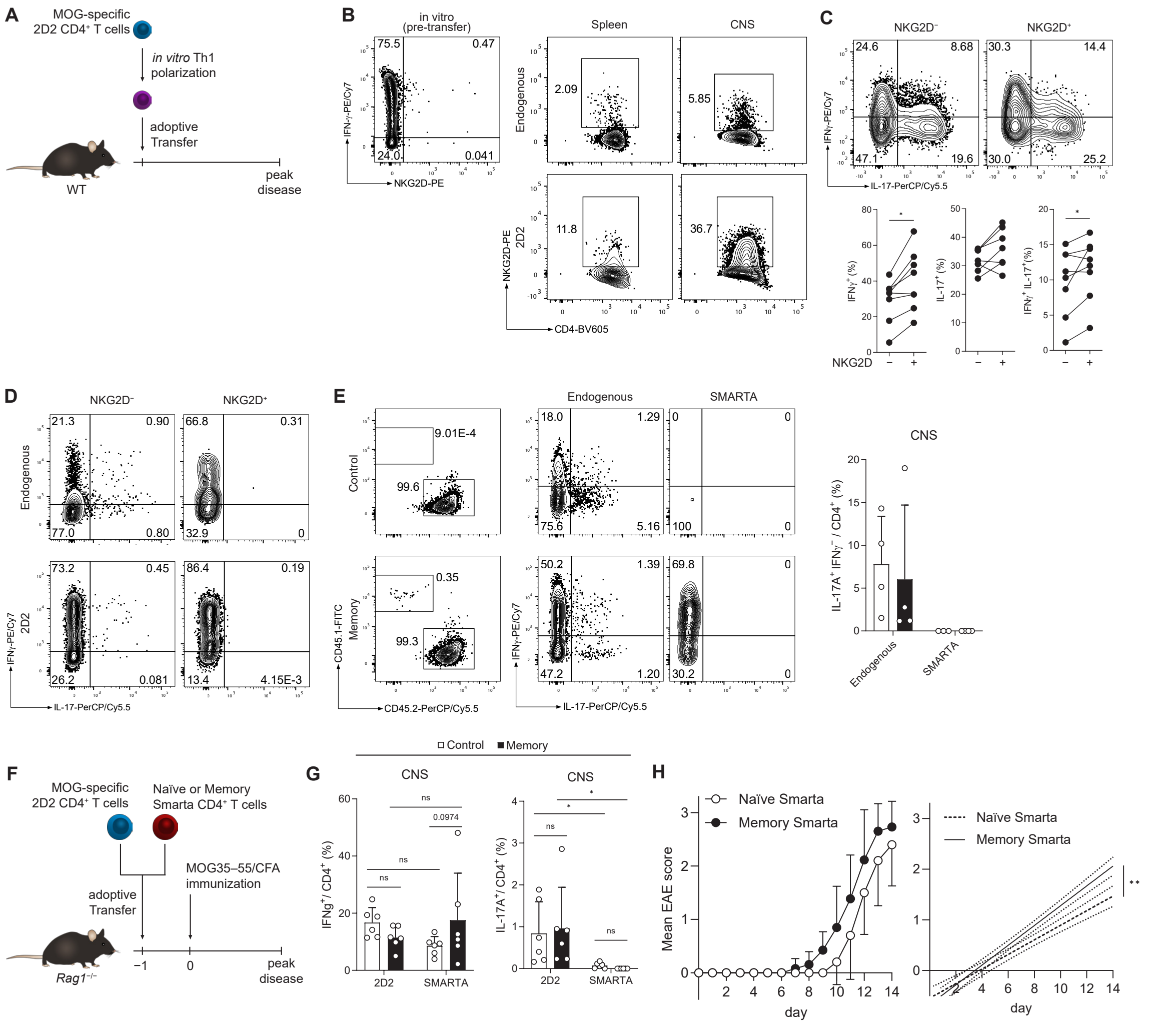

Fig. S6. Bystander activation of memory CD4$^+$ T cells in the CNS in EAE. A, experimental layout of the 2D2 cell-mediated EAE model. B, *in vitro* differentiated Th1 2D2 cells were analyzed for expression of NKG2D and IFN-γ before transfer for EAE induction (left). At the peak of disease, CD4$^+$ T cells were analyzed for expression of NKG2D (right). C, NKG2D, IFN-γ and IL-17 expression in CD4$^+$ T cells isolated from the CNS of MOG$_{35\text{-}55}$/CFA-immunized B6 mice (n = 7–8). D, CD4$^+$ T cells isolated from the CNS of Th1 adoptive transfer EAE mice were analyzed for expression of IFN-γ and IL-17. E, MOG$_{35\text{-}55}$/CFA-immunized B6 mice were injected with either 2x10^6 naïve or memory SMARTA cells. SMARTA (CD45.1$^+$CD45.2$^-$) and endogenous (CD45.1$^-$CD45.2$^+$) CD4$^+$ T cells from the CNS were analyzed for expression of IFN-γ and IL-17 (n = 4). Representative plots and summary graphs are shown. F-H, 5x10^5 Smarta + 5x10^5 2D2 were transferred into *Rag1$^{-/-}$* mice for EAE induction. Experimental layout (F), cytokine production (G) and clinical scores with linear regression (H, 95% confidence interval, n=10-13). Mean ± s.d., Wilcoxon matched-pairs signed rank test (C) and two-way ANOVA (Šídák; E, G)

Table S2. Antibodies used for CyTOF analysis.

Target antigen	Antibody clone	Label	Catalog number
CD45	HI30	089Y	Fuidigm 3089003B
Ly6G/C (Gr1)	RB6-8C5	141Pr	Fuidigm 3141005B
TIGIT	1G9	143Nd	Biolegend 142102
IL-2	JES6-5H4	144Nd	Fuidigm 3144002B
CD69	H1.2F3	145Nd	Fuidigm 3145005B
CD8a	53-6.7	146Nd	Fuidigm 3146003B
NKG2D (CD314)	CX5	147Sm	Biolegend 130204
CD11b (MAC1)	M1/70	148Nd	Fuidigm 3148003B
CD19	6D5	149Sm	Fuidigm 3149002B
CD49d	R1-2	151Eu	Fuidigm 3151016B
CD3e	145-2C11	152Sm	Fuidigm 3152004B
CD226	10E5	153Eu	Biolegend 128822
CD155	TX56	155Gd	Biolegend 131502
CD90	30-H12	156Gd	Fuidigm 3156006B
FoxP3	FJK-16s	158Gd	Fuidigm 3158003A
CD279 (PD1)	29F.1A12	159Tb	Fuidigm 3159024B
CD62L	MEL-14	160Gd	Fuidigm 3160008B
CD96	3.3	161Dy	Biolegend 131704
CD366 (TIM3)	RMT323	162Dy	Fuidigm 3162029B
IL-10	JES5-16E3	163Dy	Biolegend 505012
CD49b	HMa2	164Dy	Fuidigm 3164011B
IFN-γ	XMG1.2	165Ho	Fuidigm 3165003B
ICOS	C398.4A	168Er	Fuidigm 3168024B
TCRβ	H57-597	169Tm	Fuidigm 3169002B
NK1.1	PK136	170Er	Fuidigm 3170002B
CD44	IM7	171Yb	Fuidigm 3171003B
CD4	RM4-5	172Yb	Fuidigm 3172003B
CD223 (LAG3)	C9B7W	174Yb	Fuidigm 3174019B
CD127	A7R34	175Lu	Fuidigm 3175006B
B220	RA3-6B2	176Yb	Fuidigm 3176002B
CD11c	N418	209Bi	Fuidigm 3209005B

Dataset S1 (separate file). Sequencing data are deposited on the ArrayExpress database at EMBL-EBI is available vis the accession number E-MTAB-11521.
